# Single-cell evaluation reveals shifts in the tumor-immune niches that shape and maintain aggressive lesions in the breast

**DOI:** 10.1038/s41467-021-25240-z

**Published:** 2021-08-18

**Authors:** Vidya C. Sinha, Amanda L. Rinkenbaugh, Mingchu Xu, Xinhui Zhou, Xiaomei Zhang, Sabrina Jeter-Jones, Jiansu Shao, Yuan Qi, John A. Zebala, Dean Y. Maeda, Florencia McAllister, Helen Piwnica-Worms

**Affiliations:** 1 Department of Experimental Radiation Oncology, The University of Texas MD Anderson Cancer Center, Houston, TX 77030 USA; 2 Department of Genomic Medicine, The University of Texas MD Anderson Cancer Center, Houston, TX 77030 USA; 3 Department of Bioinformatics and Computational Biology, The University of Texas MD Anderson Cancer Center, Houston, TX 77030 USA; 4 Syntrix Pharmaceuticals, Inc., Auburn, WA 98001 USA; 5 Department of Clinical Cancer Prevention, The University of Texas MD Anderson Cancer Center, Houston, TX 77030 USA

**Keywords:** Breast cancer, Cancer microenvironment, Tumour heterogeneity, Tumour immunology, Experimental models of disease

## Abstract

There is an unmet clinical need for stratification of breast lesions as indolent or aggressive to tailor treatment. Here, single-cell transcriptomics and multiparametric imaging applied to a mouse model of breast cancer reveals that the aggressive tumor niche is characterized by an expanded basal-like population, specialization of tumor subpopulations, and mixed-lineage tumor cells potentially serving as a transition state between luminal and basal phenotypes. Despite vast tumor cell-intrinsic differences, aggressive and indolent tumor cells are functionally indistinguishable once isolated from their local niche, suggesting a role for non-tumor collaborators in determining aggressiveness. Aggressive lesions harbor fewer total but more suppressed-like T cells, and elevated tumor-promoting neutrophils and IL-17 signaling, disruption of which increase tumor latency and reduce the number of aggressive lesions. Our study provides insight into tumor-immune features distinguishing indolent from aggressive lesions, identifies heterogeneous populations comprising these lesions, and supports a role for IL-17 signaling in aggressive progression.

## Abstract (web summary)

The classification of breast lesions as indolent or aggressive to tailor treatment is crucial. Here, the authors use single-cell transcriptomics and multiparametric imaging of a breast cancer mouse model, report distinct tumor-immune features for the two types of lesions, and suggest the role of IL-17 signaling in disease progression.

## Introduction

Ductal carcinoma in situ (DCIS) is a non-invasive lesion of the breast that is thought to serve as a non-obligate precursor of invasive breast cancer. DCIS now comprises ~15–30% of newly diagnosed breast cancers in the United States^1–3^. Currently, almost all DCIS patients are treated indiscriminately with surgical resection of the lesion, with or without adjuvant endocrine or radiation therapy, an approach unlikely to be optimal for most patients^4^. Unfortunately, it is currently not possible to differentiate lesions that will progress to invasive, potentially lethal disease from those that will remain indolent (and thus could be spared treatment), underscoring the clinical need for consensus stratification of breast lesions as indolent or aggressive to guide treatment. Indeed, several clinical studies are underway to address these urgent questions^5–11^.

Improvement in the clinical management of DCIS is limited by our incomplete understanding of early breast cancer biology. Studies on intraepithelial breast neoplasias have been limited by the technical challenges of isolating small lesions from adjacent normal tissue and obtaining sufficient tumor material for downstream analyses^12^. Nonetheless, valuable insight from these studies has cumulatively led to at least two major, non-mutually exclusive schools of thought to explain the differences between indolent and aggressive disease. One hypothesis focuses primarily on changes that occur intrinsically within transformed epithelial cells as they break through the basement membrane, while another emphasizes a major role for a distorted lesion niche that switches from inhibiting invasion to permitting (and even promoting) it^13–20^.

Recently, studies have evaluated early-stage breast lesions using single-cell approaches assaying both genotype^21,22^ and phenotype^23^, allowing insights into in situ lesion biology that may have been partly obscured by bulk analyses. However, a single-cell transcriptional view of intraepithelial breast lesions is currently lacking, and the heterogeneous transcriptional subpopulations that comprise indolent and aggressive lesions are undefined.

Here, to identify features that distinguish indolent and aggressive lesions and to identify potential determinants of lesion aggressiveness, we use an ERBB2 mouse model of breast cancer that generates both indolent (in situ, slow growing) and aggressive (invasive, rapidly growing) lesions within the same mammary gland. We interrogate the tumor and immune niche compartments of both indolent and aggressive lesions, and functionally measure the degree to which these features shape lesion behavior, using a combination of single-cell transcriptomic analyses, multiparametric imaging, and functional studies. Our work provides insight on the heterogeneous transcriptional and phenotypic populations that comprise these lesions, supports a role for IL-17 signaling in mammary lesion progression, and generates a collection of tumor and immune niche features that may be useful in identifying lesions that are capable of aggressive behavior, including those that might be otherwise considered indolent.

## Results

### Aggressive progression occurs in a minority of caErbB2-expressing breast lesions

**Fig. 1:**
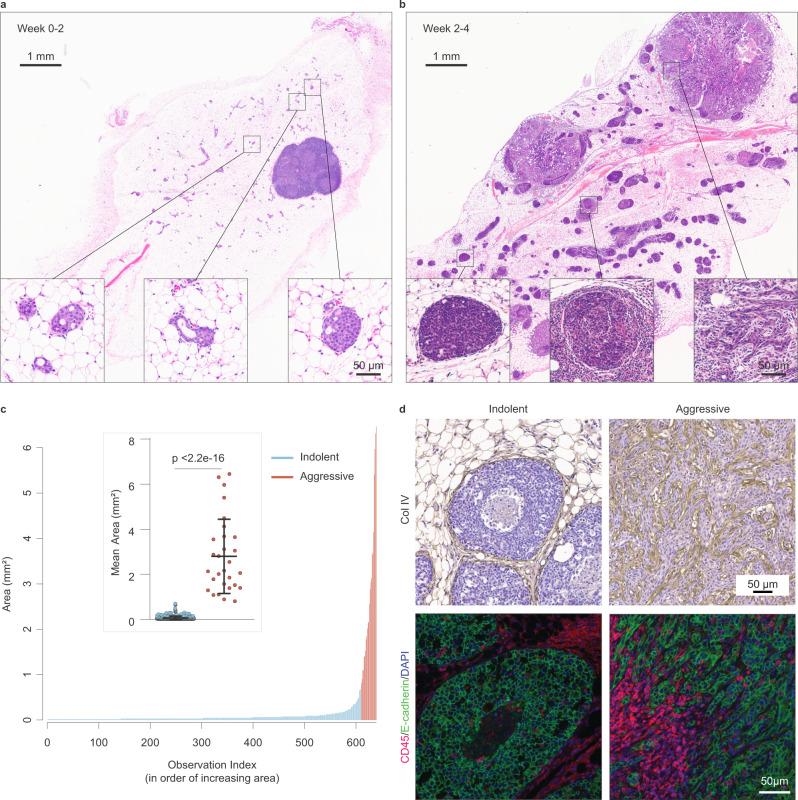
Aggressive progression occurs in a minority of breast lesions and is associated with heavy immune infiltration. **a**, **b** Mouse model of breast cancer exhibits the progression of mammary lesions over time. Mammary glands were collected from animals at various time points following intraductal injection and stained by hematoxylin & eosin (H&E) to visualize lesions at 0–2 weeks (**a**) or 2–4 weeks (**b**) after injection. High-power magnification (inset) shows in situ lesions of early time points (**a**), and a combination of in situ (indolent; **b,** left inset) and invasive (aggressive; **b**, right inset) lesions at later time points. Lesions exhibiting a well-circumscribed tumor compartment with heavy stromal involvement (**b**, center inset) were also observed. **c** Quantification of lesions present 2–4 weeks after injection reveals minority of rapidly progressing large lesions. Mammary glands bearing a combination of indolent and aggressive lesions were evaluated for individual lesion size. Index plot shows all lesions quantified across five animals. Inset dot plot shows mean area of top 5% largest lesions vs. remainder. *P* value (<2.2e−16) was calculated using two-sided Wilcoxon rank sum test for non-normal data; center line, mean; error bars, standard deviation (SD), *U* = 0. **d** Aggressive lesions are heavily immune infiltrated. Lesion-bearing glands were immunostained for collagen IV (top panel) and E-cadherin/CD45 (bottom panel) to assess, respectively, integrity of basement membrane and associated immune infiltration of indolent and aggressive lesions. Representative results from *n* = 3 independent animals. See also Supplementary Fig. 1. Source data are provided as a Source Data file.

To model early stages of tumor initiation, we delivered lentiviral particles carrying constitutively activated rat *Erbb2* tagged with HA and GFP (caErbB2)^24^ through the lactiferous glands of adult virgin female FVB mice^25,26^. The resulting caErbB2-initiated lesions within the same mammary gland progressed asynchronously, as evidenced by the simultaneous presence of lesions at various stages of advancement (Fig. 1a, b; Supplementary Fig. 1a). A minor population (~5–10%) of lesions grew extremely rapidly such that, beyond ~1 mm^2^, these lesions were significantly larger (30–40-fold; *p* < 0.0001) than the remaining 90–95% of lesions (Fig. 1c). Based on this asynchronous progression, we inferred that the majority of lesions progressed at a relatively indolent pace and remained small, while a minority advanced aggressively to form large tumors.

Histopathological evaluation of lesion-bearing mammary glands confirmed that while the majority of indolent lesions remained constrained by an intact basement membrane, aggressive lesions consistently exhibited widespread invasion beyond the confines of the duct (Fig. 1d, top panel; Supplementary Fig. 1b). Immunostaining of the basement membrane protein collagen IV not only confirmed a loss of basement membrane integrity in aggressive lesions, but also revealed extremely irregular and intratumoral expression of collagen IV. Although indolent and aggressive lesions both classified as Luminal B based on HER2, Ki67, and estrogen receptor (ER) expression^27^ (Supplementary Fig. 1c–e), aggressive lesions often exhibited spindloid and squamous metaplastic tumor cells, with the latter frequently adjacent to keratin-like structures or keratin pearls (Supplementary Fig. 1a). Finally, we observed that indolent and aggressive lesions were each associated with distinct local microenvironments. Most notably, aggressive lesions were associated with a heavy immune infiltrate compared to indolent lesions (Fig. 1d, bottom panel).

Despite lesions in the same animal experiencing the same initiating oncogene (active *Erbb2*), only a relatively rare subset of these lesions gained the functional capacity to progress aggressively or recruit immune cells. These observations strongly suggest that additional factors cooperated to determine the likelihood and rate of lesion progression, and that only a relatively rare subset of these lesions gained the functional capacity to progress aggressively. Thus, this model system provides a unique opportunity to distinguish lesions that progress rapidly from those that progress at a relatively indolent pace, and to identify potential cell-intrinsic and extrinsic determinants of aggressive lesion progression.

### Tumor cells from indolent vs. aggressive lesions are transcriptionally distinct

**Fig. 2:**
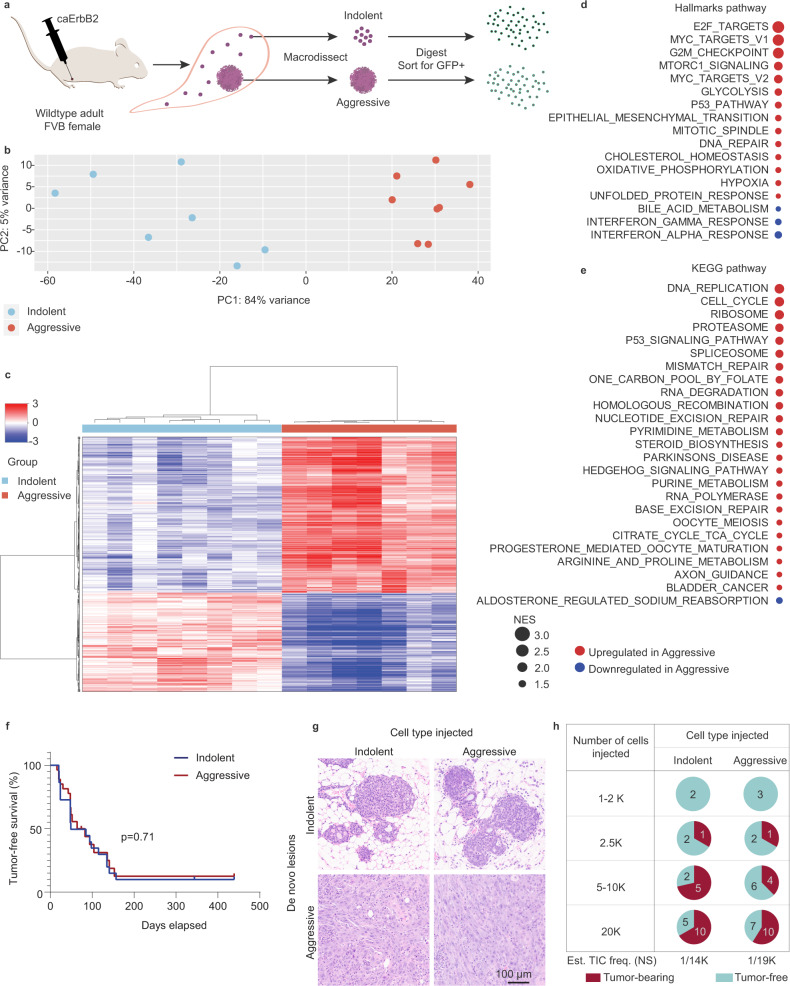
Tumor cells from indolent vs. aggressive lesions are transcriptionally distinct, but functionally indistinguishable in a new host. **a** Schema of bulk RNA-sequencing assay. Lesion samples were pooled from 2 to 5 animals per sample (21 total animals) to acquire sufficient material for analysis. **b** Principle component analysis of indolent vs. aggressive samples. **c** Heatmap of differentially expressed (DE) genes (FDR < 0.05) in indolent vs. aggressive samples. Values indicate median-centered variance-stabilizing-transformed counts. Normalized enrichment scores of Hallmarks (**d**) and KEGG (**e**) pathways in aggressive lesions vs. indolent following gene set enrichment analysis (GSEA). Pathways with FDR < 0.05 shown in order of normalized enrichment score (NES). Red and blue NES indicate, respectively, positive and negative enrichment in aggressive samples. **f** Kaplan–Meier plot showing tumor-free survival curves of animals injected intraductally with indolent or aggressive tumor cells. Two-sided p value calculated using log-rank (Mantel–Cox) test. Indolent group, *n* = 22; aggressive group, *n* = 27. **g** H&E of de novo lesions arising in the host after intraductal injection of indolent or aggressive tumor cells. Representative results from *n* = 18 animals per group. **h** Proportion of animals bearing tumors or remaining tumor-free ~400 days following injection of limiting dilutions of indolent or aggressive tumor cells. Number evaluated per group and dilution indicated in pie. Estimated tumor-initiating cell frequency (Est. TIC freq.) calculated using Extreme Limiting Dilution Analysis. NS non-significant, *p* value > 0.05. See also Supplementary Fig. 2, Supplementary Tables 1 and 2. Source data are provided as a Source Data file.

To more thoroughly characterize cell-intrinsic differences between indolent and aggressive lesions, bulk RNA sequencing (RNA-seq) was performed on tumor cells isolated from indolent and aggressive lesions (Fig. 2a, Supplementary Fig. 2a, Supplementary Data 1).

Transcriptional analysis of these tumor cells revealed that indolent and aggressive lesions were distinct from one another, as determined by principal component analysis (PCA) (Fig. 2b), and differential gene expression (DE) analysis, which identified over 8000 differentially expressed genes (8209 DE at false discovery rate (FDR) < 0.05; 1360 DE at FDR < 0.05, fold change >2; Fig. 2c, Supplementary Fig. 2b, c). Gene set enrichment analysis (GSEA) showed significant differences in several key cancer-associated pathways (Fig. 2d, e; Supplementary Tables 1 and 2). In particular, aggressive lesions exhibited enrichment of cell cycle and DNA replication pathways (Fig. 2e), as well as E2F- and Myc-associated Hallmark pathways (Fig. 2d). Furthermore, genes associated with DNA repair, G2/M checkpoint, p53 signaling, and metabolic (oxidative phosphorylation, glycolysis) pathways were also increased (Fig. 2d, e), indicative of elevated oncogenic, replicative, and metabolic stress known to accompany rapid tumor growth and progression^28^. Aggressive lesions also overexpressed EMT pathway genes (Fig. 2d), consistent with reports that invasiveness is facilitated by the transition of epithelial cells to a more mesenchymal state^29–31^. These findings also confirmed our own histopathological observations of spindloid and squamous metaplastic cells in aggressive lesions. In addition, aggressive lesions exhibited negative enrichment of immune-associated pathways (Fig. 2d) despite heavy immune infiltration into these lesions (Fig. 1d, bottom panel), suggesting that aggressive tumor cells may be capable of dampening anti-tumor immunity. Notably, aggressive lesions expressed ERBB family members at comparable (*Erbb2* and *Egfr)* or lower (*Erbb3* and *Erbb4*) levels compared to indolent lesions (Supplementary Fig. 2d–g), and also did not display gene set enrichment for the ERBB signaling pathway (Supplementary Fig. 2h), suggesting that aggressive lesions may be less dependent on ERBB signaling for continued aggressive behavior. Finally, to evaluate the intrinsic subtypes of breast cancer(s) most represented by our model, we performed PAM50 analysis on the transcriptional profiles of indolent and aggressive lesions^32^. In contrast to St. Gallen surrogate subtyping, PAM50 analysis revealed that our indolent lesions were most transcriptionally similar to Normal-like and Luminal A human subtypes, while aggressive lesions resembled Basal-like human subtypes (Supplementary Fig. 1f).

Taken together, the transcriptional profile of large, rapidly progressing lesions is consistent with aggressive growth, activation of developmental pathways leading to epithelial metaplasia, and inhibition of anti-tumor immune responses that might have otherwise limited tumor growth and progression.

### Cell-intrinsic features are insufficient to drive aggressive lesion behavior

To ascertain the degree to which aggressive lesion behavior was determined by cell-intrinsic features, we tested the lesion-forming capacity of epithelial cells isolated from both indolent and aggressive lesions. We hypothesized that if lesion behavior was driven primarily by cell-intrinsic properties, then cells from indolent lesions would give rise to slow growing, in situ lesions, while cells from aggressive lesions would give rise to rapidly progressing, invasive lesions. To test this hypothesis, we isolated GFP+ tumor cells from indolent and aggressive lesions and immediately re-injected them into the mammary ducts of wild type, non-tumor-bearing adult female mice to test their ability to give rise to new lesions. Surprisingly, we found that indolent and aggressive lesions were indistinguishable from one another in their ability to initiate new tumors when placed in matched microenvironments of new, non-tumor-bearing hosts (Fig. 2f). In particular, we found that cells derived from indolent lesions were capable of forming both in situ and invasive lesions, as were those derived from aggressive lesions (Fig. 2g). In addition, injection of a limiting dilution of cells revealed no significant difference in lesion-forming capacity of indolent vs. aggressive lesions calculated using extreme limiting dilution analysis (ELDA)^33^ (*p* = 0.34, Fig. 2h). Taken together, these results suggest that cell-intrinsic features alone were insufficient to drive aggressive behavior.

### The aggressive tumor niche harbors an expanded basal-like population

**Fig. 3:**
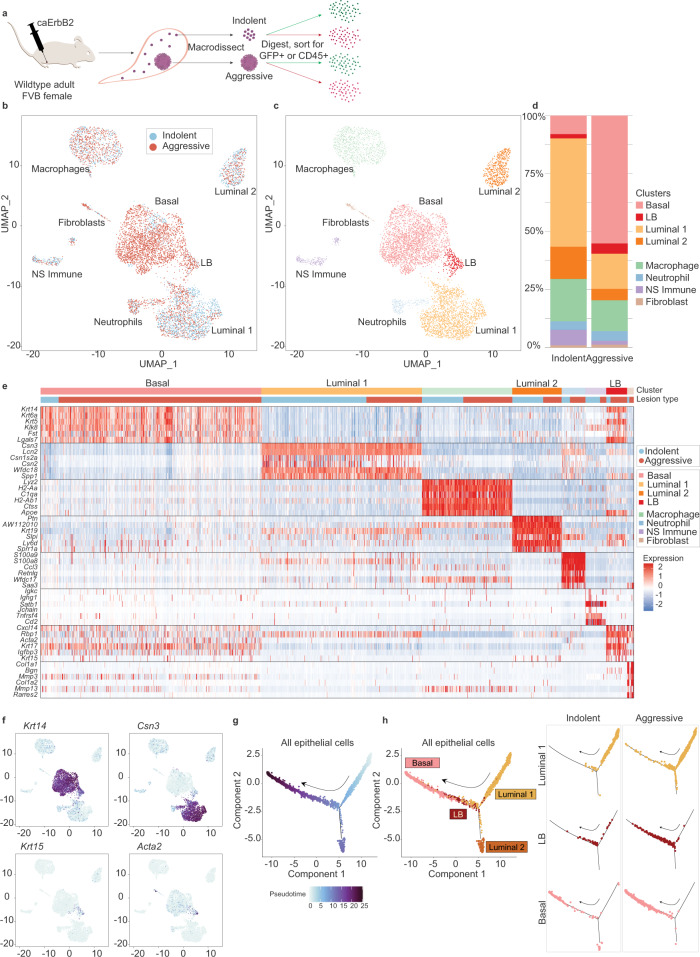
The aggressive tumor niche harbors an expanded basal-like population that is transcriptionally linked to a luminal-like population through an intermediate state. **a** Schema of single-cell RNA sequencing assay. Four samples (indolent tumor cells, indolent immune cells, aggressive tumor cells, and aggressive immune cells) were obtained from 13 pooled mice to acquire sufficient material for analysis. **b**–**d** Uniform Manifold Approximation and Projection (UMAP) was used for dimension reduction of single-cell data. Tumor fractions from indolent and aggressive lesions were computationally merged (**b**) for cluster analysis and identification (**c**). Proportion of clusters per group shown in **d**. **e** Heatmap of top differentially expressed (DE) genes for each cluster, ordered in decreasing cluster size. Clusters indicated by top horizontal bar, with inferred tumor clusters noted (Basal, Luminal 1, Luminal 2, LB). Cells derived from indolent or aggressive tumor sample indicated by second horizontal bar. DE genes are listed on the left. **f** UMAP plots of all tumor cells, colored by expression of *Krt14* for basal-like cells, *Csn3* for Luminal 1 cells, *Krt15*, and *Acta2* for LB cells. Trajectory plot of all tumor cells, colored by pseudotime value (**g**) and cluster (**h**, left panel). Luminal 1, LB, and basal cells were plotted by lesion type to visualize distribution along trajectory of cells from indolent vs. aggressive tumors (**h**, right panel). Arrow indicates increasing pseudotime value. See also Supplementary Fig. 3. Source data are provided as a Source Data file.

**Fig. 4:**
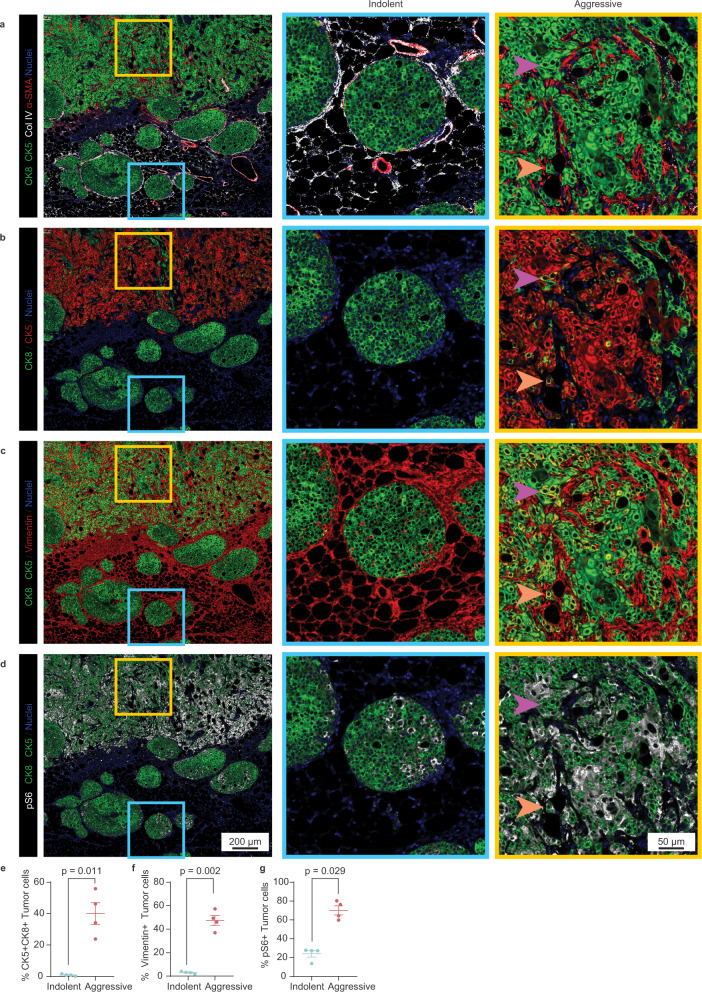
Multiparametric imaging of indolent and aggressive lesions reveals spatially heterogeneous tumor and immune niches. **a**–**d** All images shown are pseudo-colored channels from a single mammary tissue section bearing indolent and aggressive lesions stained using a cocktail of antibodies and visualized using imaging mass cytometry. Lesions were stained for collagen IV (basement membrane), α-SMA (smooth muscle actin, myoepithelial marker), cytokeratin 8 (luminal epithelial marker), cytokeratin 5 (basal epithelial marker), vimentin (mesenchymal marker), and pS6 (S235/S236, mTOR signaling). Combinations of markers were selected to evaluate indolent and aggressive lesions for membrane integrity (**a**), epithelial identity and mixed epithelial lineage (**b**), mixed epithelial/mesenchymal lineage (**c**), and metabolic activity (**d**). Representative images on the left panel show low-magnification view of adjacent indolent and aggressive lesions. Boxes in left panel correspond by color to high magnification images, shown in middle (blue, indolent), and right (yellow, aggressive) panels. Arrows of the same color point to the same cell in each image. Quantification of indolent or aggressive tumor cells co-expressing CK5 and CK8 (**e**), vimentin (**f**), and pS6 (**g**). *P* value was calculated using two-tailed Welch’s t-test (**e**, *t* = 5.588, degrees of freedom (df) = 3.012; **f**, *t* = 10.50, df = 3.075) or two-sided Wilcoxon rank-sum test (**g**, *U* = 0); error bars indicate standard error of the mean (SEM). Each dot represents quantification from one region. See also Supplementary Fig. 4. Source data are provided as a Source Data file.

To investigate how co-evolving epithelial and immune cells might functionally govern lesion aggressiveness, we performed single-cell RNA sequencing (scRNA-seq) of tumor (GFP+) and immune (CD45+) cells isolated from both indolent and aggressive lesions (Fig. 3a, Supplementary Fig. 2i, Supplementary Data 1). Analysis of the tumor epithelial cell compartment revealed two luminal-like populations (major Luminal 1 and minor Luminal 2) that comprised the bulk of indolent lesions, and a basal-like population (Basal) that comprised the bulk of aggressive lesions (Fig. 3b–f, Supplementary Fig. 3a–c). Indolent and aggressive lesions harbored significantly different proportions of tumor clusters overall and individually (Supplementary Fig. 3d–f). We also identified contaminant populations of fibroblasts and immune cells within the GFP+ fraction (Supplementary Fig. 3g), that were useful for validating our clustering approach. Immunostaining by imaging mass cytometry (Fig. 4) for luminal and basal cytokeratins (CK8 and CK5, respectively) confirmed that indolent and aggressive lesions were indeed comprised of two major distinct epithelial subtypes. Consistent with some types of human in situ lesions, indolent lesions in our model were comprised primarily of cuboidal-shaped luminal (CK8+) tumor cells, relatively uniform in appearance throughout the lesion cross-section, with a single discontinuous layer of basal (CK5+) and myoepithelial (α-SMA) cells in the surrounding basal compartment^34,35^. In contrast, invasive lesions harbored an expanded CK5+ population that was not restricted to the basal compartment and instead intermixed with CK8+ cells within the tumor bulk (Fig. 4a, b; Supplementary Fig. 4a, d).

Interestingly, single-cell analysis identified an intermediate cell population (designated LB, for Luminal-Basal) whose transcriptional profile contained features of both Luminal 1 and Basal clusters, and which expanded as lesions advanced. A similar luminal-basal mixed-lineage population was also detected histologically within invasive lesions co-expressing luminal and basal epithelial markers (CK8, CK5; Fig. 4b, E; Supplementary Fig. 4), a subset of which also expressed the mesenchymal marker vimentin (Fig. 4c, f). These cells could be identified among single positive cells (Supplementary Fig. 4a, b). Very rarely, cells that appeared to be in the process of transitioning between single and double CK positive states were observed (Supplementary Fig. 4c). The emergence of this mixed-lineage population, in addition to the broad presence of tumor cell metaplasia within the aggressive niche, suggests that rapid lesion progression in this model may be associated with loss of epithelial identity, increased plasticity, and/or expansion of precursor populations^36^.

To visualize the degree of transcriptional relatedness between tumor cells, we constructed pseudotemporal trajectories by Monocle v2^37,38^. As suggested by our clustering analysis, we found a transcriptional continuum between Luminal 1 cells and Basal cells, through LB intermediates (Fig. 3g, h). In contrast, Luminal 2 cells were found to be relatively transcriptionally distinct from other epithelial populations (Fig. 3h, left panel). Luminal 1, LB, and Basal cells from indolent and aggressive lesions were visualized individually to identify their distributions on the trajectory (Fig. 3h, right panel). Cells from aggressive lesions tended to be distributed more broadly along the trajectory and, in particular, aggressive Luminal 1 and LB populations projected further towards the Basal point of the trajectory, compared to indolent cells of the same cluster. These distributions suggest that aggressive Luminal and LB clusters harbor greater transcriptional heterogeneity and Basal-like features compared to their indolent counterparts. Inferred copy-number analysis using inferCNV^39^ revealed a minority of Basal cells with inferred gains (Supplementary Fig. 3h, i), and more aneuploid chromosomes than all other populations (Supplementary Fig. 3j, k), in agreement with a model in which normoploid Luminal 1 cells represent an early stage in tumor progression, while Basal cells represent a more advanced, aneuploid stage. Taken together these data suggest that some breast cancer cells may undergo a cell-state transition between luminal-like and basal-like phenotypes, through an identifiable intermediate transition state, during progression from in situ to invasive disease.

### Tumor subpopulations exhibit heterogeneous pathway enrichment that become more pronounced in aggressive lesions

**Fig. 5:**
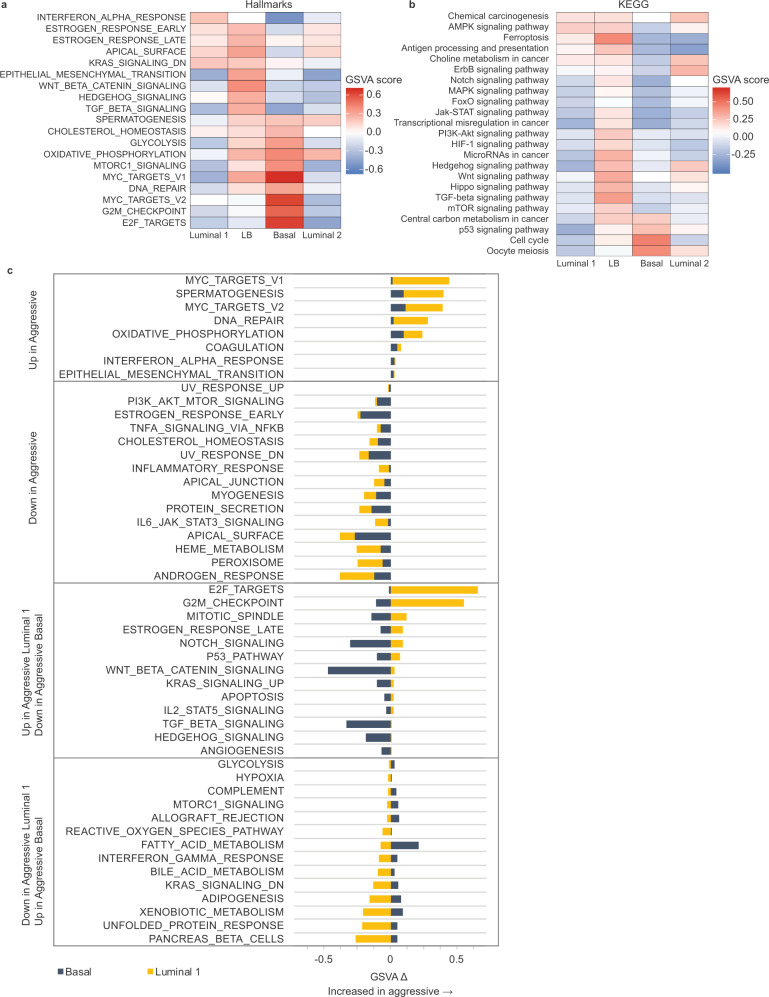
Heterogeneous pathway enrichment across tumor subpopulations suggest distinct functional roles in the tumor niche. **a**, **b** Comparison of pathway enrichment across tumor subpopulations. Heatmap of enrichment scores of selected Hallmarks (**a**) and KEGG (**b**) pathways in tumor clusters following gene set variation analysis (GSVA). Positive score indicates relative enrichment vs. other populations identified by scRNA-seq and pathways within the same collection. Cells from indolent and aggressive lesions were analyzed together per cluster. **c** Comparison of pathway changes in indolent vs. aggressive lesions: clusters were analyzed by lesion type to calculate a change in Hallmark pathway enrichment score (GSVA delta, Δ) in aggressive vs. indolent lesions. Positive score indicates increased score in aggressive lesions. Pathways were classified as concordantly (top two panels) or discordantly (bottom two panels) shifted in Luminal 1 and Basal populations of aggressive lesions compared to indolent lesions. Source data are provided as a Source Data file.

Given the shift in frequencies of different tumor cell types in aggressive and indolent lesions, we next asked whether these different tumor cell populations exhibited detectable differences, beyond cell identity, in their imputed function. Evaluation of Hallmark and KEGG gene signatures by gene set variation analysis (GSVA, Fig. 5a, b) largely aligned with differences identified by bulk transcriptomic profiling of indolent and aggressive tumor cells. The ERBB2 signaling pathway (KEGG) was enriched within the Luminal populations, which was expected given the oncogenic event introduced into these cells was caErbB2. Notably, the Basal population exhibited a relative decrease in these pathways, suggesting that these cells may be less reliant on ERBB2 signaling for continued oncogenic proliferation or progression (Fig. 5a, b).

Instead, the Basal population showed enrichment of E2F and Myc signaling (Hallmarks), cell cycling (KEGG), G2/M checkpoint (Hallmarks), and p53 signaling (KEGG) pathways, consistent with proliferation, growth, and replicative stress (Fig. 5a, b), as seen in bulk aggressive lesions (Fig. 2d, e). Relative enrichment of spermatogenesis (Hallmarks) and oocyte meiosis (KEGG) pathways suggests the inappropriate somatic upregulation of meiotic genes associated with disrupted mitosis^40–43^. As might be expected for rapidly cycling cells, the Basal population also exhibited enrichment of metabolic pathways (Fig. 5a, b), validated by increased levels of phosphorylated-S6 (Fig. 4d, g).

Consistent with our hypothesis that the LB population represents a mixed-lineage state, this population exhibited increased expression of genes associated with developmental and embryonic programs (Fig. 5a, b), including Wnt-beta-catenin, Hedgehog, TGF-beta, and Notch, and Hippo signaling, as well epithelial-to-mesenchymal transition (Hallmarks, KEGG).

Interestingly, we observed a modest enrichment of antigen processing and presentation (KEGG) and interferon-alpha signaling (Hallmarks) in the Luminal 1 population (Fig. 5a, b). In contrast, the relative decrease of these pathways in the Basal population suggests that cells in the aggressive niche may be more resistant to immune surveillance, perhaps permitting their rapid expansion.

To identify whether Luminal 1 and Basal cells shift their function based on lesion aggressiveness, we analyzed these populations by lesion type and calculated the change in GSVA pathway enrichment scores in aggressive lesions relative to indolent lesions. We identified pathways for which both Luminal 1 and Basal populations were increased (e.g., Myc signaling) and decreased (e.g., apical surface) (Fig. 5c, top panels) in aggressive lesions relative to indolent lesions. We also identified a set of pathways that were discordantly altered in aggressive populations. Specifically, oncogenic signaling and DNA stress pathways were increased in luminal but not basal populations in aggressive lesions relative to indolent lesions, whereas metabolic pathways were increased in basal but not luminal populations in aggressive lesions relative to indolent lesions (Fig. 5c, bottom panels). Opposing pathway changes suggest that these tumor cell populations may play increasingly divergent functional roles within the lesion niche.

### The aggressive niche harbors an expanded and spatially restricted neutrophil population

**Fig. 6:**
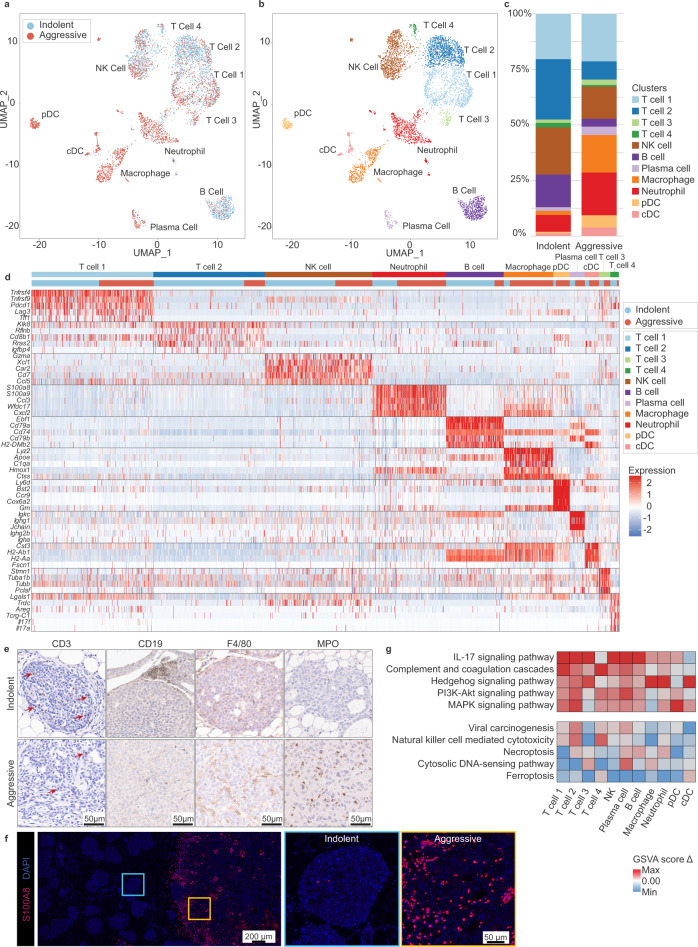
The aggressive niche harbors an expanded and spatially restricted myeloid population. **a**–**c** UMAP was used for dimension reduction of single-cell data. Immune fractions from indolent and aggressive lesions were computationally merged (**a**) for cluster analysis and identification (**b**). Proportion of clusters per group shown in **c**. NK natural killer, pDC plasmacytoid dendritic cell, cDC conventional dendritic cell. **d** Heatmap of top differentially expressed (DE) genes for each cluster, ordered in decreasing cluster size. Clusters indicated by top horizontal bar, with inferred immune identity noted. Cells derived from indolent or aggressive tumor sample indicated by second horizontal bar. DE genes are listed on the left. **e** Immunohistochemical staining for major lymphoid (T cells—CD3; B cells—CD19) and myeloid (macrophages—F4/80; neutrophils—MPO) populations associated with indolent and aggressive lesions. Arrows point to selected positively stained cells. Representative images shown from *n* = 4, 3, 3, 4 animals for CD3, CD19, F4/80, and MPO, respectively. **f** Immunofluorescent staining for neutrophils using an alternative marker S100A8. Left panel shows low power magnification view of adjacent indolent and aggressive lesions, with S100A8+ infiltration tightly localized with the aggressive niche. Boxes correspond by color to high magnification images shown middle (blue, indolent) and right (yellow, aggressive) panels. Representative images shown from *n* = 7 animals. **g** Immune clusters were analyzed by lesion type to calculate a change in KEGG pathway enrichment score (GSVA delta) in aggressive vs. indolent lesions. Positive score indicates increased enrichment score in aggressive lesions. Top 5 pathways most commonly increased or decreased across populations were identified to ascertain potential niche-wide changes associated with aggressive lesions. See also Supplementary Figs. 5–7. Source data are provided as a Source Data file.

To characterize the immune composition of indolent and aggressive lesions, we evaluated scRNA-seq data acquired from CD45+ cells sorted by FACS from these two lesion types. We identified major immune cell types, including T cells (4 clusters), B cells, plasma cells, NK cells, neutrophils, macrophages, and dendritic cells (Fig. 6a, b, d; Supplementary Fig. 5a, b). The detection of a broad range of immune cell types in our model is consistent with previous findings in human breast cancer^44–48^. While these immune cell populations were identified in both lesion types, their relative frequencies were shifted (Fig. 6c; Supplementary Fig. 5c–e). To validate these findings, we performed immunostaining for several lymphoid and myeloid cells, including T cells, B cells, macrophages, and neutrophils (Fig. 6e). In agreement with our scRNA-seq data, immunostaining showed decreased lymphoid cells (T cells, B cells) but increased myeloid cells (macrophages, neutrophils) in aggressive lesions (Fig. 6c, e). These findings point to a lymphoid-to-myeloid shift consistent with the reported role of myeloid populations in mediating T cell suppression. Indeed, although fewer infiltrating CD4+ and CD8+ T cells per tumor area were observed in aggressive lesions relative to indolent lesions (Supplementary Fig. 6a–d), subset analysis of T cell clusters (T cell 1–4) revealed that immune-suppressed T cell populations (identified using exhaustion scores calculated from the expression of genes associated with immune suppression) were increased in relative frequency in aggressive lesions (Supplementary Fig. 6e–h).

Validation by immunostaining for S100A8 (elevated in neutrophils and myeloid-derived suppressor cells^49,50^) showed that, among immune cells identified by staining, S100A8+ neutrophils exhibited the greatest dichotomy between indolent and aggressive niches (Fig. 6f). Invasive niches exhibited a massive infiltration of S100A8+ neutrophils, while in situ lesions were almost completely devoid of these cells. Remarkably, the exclusion of S100A8+ neutrophils from in situ lesions was maintained even when these lesions were adjacent to their invasive counterparts, suggesting that the recruitment of these cells is tightly linked to the local aggressive niche.

Consistent with the active recruitment of neutrophils, we noted that one T cell population (T cell 4, potentially gamma-delta T cells based on relatively increased expression of genes encoding gamma (*Tcrg-C1*) and delta (*Trdc*) T cell receptors, Fig. 6d) displayed relatively higher expression of genes encoding IL-17 ligands, *Il17a* and *Il17f* (Fig. 6d, Supplementary Fig. 7a, b). IL-17 is known to activate downstream signaling cascades ultimately leading to neutrophil recruitment. Genes encoding cognate receptors *Il17ra* and *Il17rc* were relatively increased, respectively, in immune cells (particularly neutrophils and macrophages, Supplementary Fig. 7c, d) and Basal tumor cells (Supplementary Fig. 7e, f). In addition, target genes downstream of IL-17 signaling (KEGG) was the most commonly increased pathway across multiple immune populations in aggressive lesions relative to indolent lesions (Fig. 6g). These data identify a niche interaction in which IL-17 cytokines produced by one population may effect signaling pathways in potential niche collaborators. Altogether, our single-cell and histological findings suggest potential roles for IL-17 signaling and/or neutrophils in distinguishing indolent from aggressive lesions, and potentially driving or supporting aggressive lesion progression.

### Some indolent lesions bear tumor and immune features associated with aggressive lesion behavior

Although we observed largely dichotomous features when comparing indolent with aggressive lesions, we did observe a subset of smaller-sized lesions that shared properties with aggressive lesions. Specifically, some of these smaller lesions exhibited mixed-lineage cells and an expanded basal-like (cytokeratin 5) population, similar to that observed in advanced lesions (Supplementary Fig. 8a, b). In addition, we observed a few rare smaller-sized lesions with accompanying neutrophils (Supplementary Fig. 8c, d). These observations suggest that the subset of indolent lesions exhibiting these features may be on their way to becoming aggressive lesions.

### Inhibition of granulocyte recruitment reduces aggressive niche formation and delays tumor progression

**Fig. 7:**
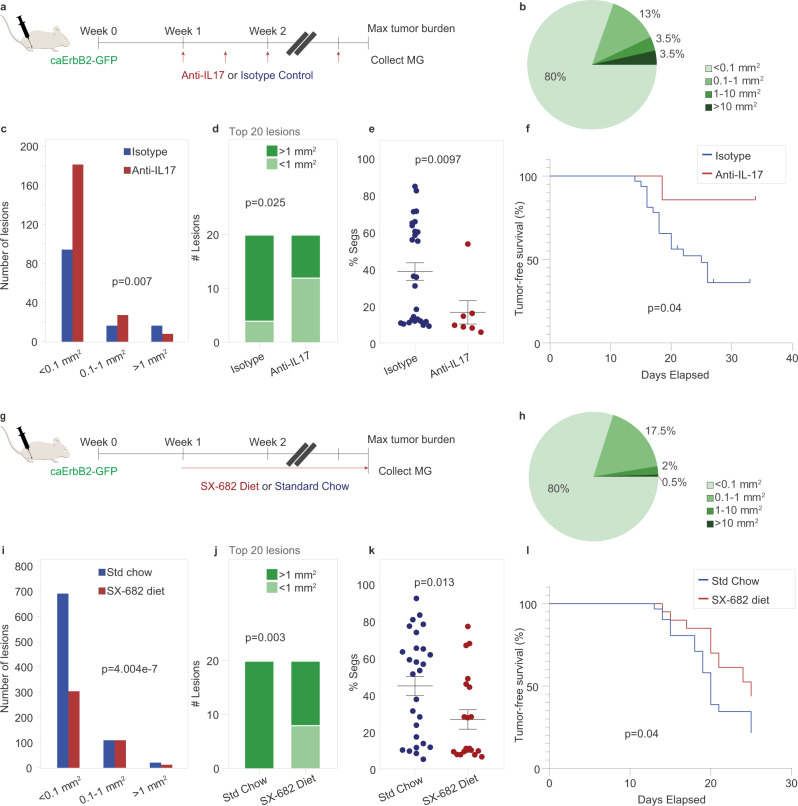
Inhibition of IL-17 and granulocyte recruitment reduces aggressive niche formation and delays tumor progression. Animals were treated with anti-IL-17/17RA or isotype control (**a**–**f**), and SX-682 diet or standard chow (**g**–**l**). **a**, **g** Schema showing experimental timeline: animals were allowed to form lesions for 1 week then treated until endpoint. All animals were collected when any one animal reached maximum tumor burden. Quantification of lesion area from animals treated with anti-IL-17/17RA or isotype control (**b**–**d**), treated in the same cohort, and SX-682 diet or standard chow (**h**–**j**), treated in the same cohort. Only mammary glands bearing lesions could be quantified. Anti-IL-17 study, *n* = 5 per group; SX-682 study, *n* = 8 per group. Pie chart showing proportion of lesions by size for Anti-IL-17 study (**b**) and SX-682 study (**h**). **c**, **i** Quantification of lesion area and number binned by size, with p value calculated by Pearson’s Chi-squared test, for Anti-IL-17 study (Chi-squared (*χ*^2^) = 10.014, df = 2) (**c**) and SX-682 study (Chi-squared = 29.462, df = 2) (**i**). **d**, **j** Stacked column graph of 20 largest lesions per group binned by size for anti-IL-17 study (**d**) and SX-682 study (**i**). *P* value calculated by two-sided Fisher’s exact test. **e**, **k** Percent segmented cells measured by complete blood count from peripheral blood collected at time of euthanasia and mammary gland collection. *P* value was calculated using two-sided Wilcoxon rank sum test; error bars indicate standard error of the mean (SEM). Each dot represents one animal. **e** Anti-IL-17 group, *n* = 7; control group, *n* = 32 pooled from multiple identically treated control cohorts; *U* = 43. **k** SX-682 group, *n* = 20; control group, *n* = 29 pooled from multiple identically treated cohorts, *U* = 169.5. **f**, **l** Kaplan–Meier plot showing tumor-free survival curves of treated animals. Two-sided *p* value calculated using log-rank (Mantel–Cox) test. **f** Anti-IL-17 group, *n* = 7; control group, *n* = 32 pooled from multiple identically treated cohorts. **l** SX-682 group, *n* = 20; control group, *n* = 29 pooled from multiple identically treated cohorts. Source data are provided as a Source Data file.

Given the putative activation of the IL-17 pathway in our lesions in addition to its well-documented role in neutrophil recruitment, we tested the impact of IL-17-blockade on lesion progression. We treated mice with antibodies against IL-17A and its receptor IL-17RA (herein referred to as anti-IL-17), or isotype control, 1 week following intraductal injection with lentiviral caErbB2, a time when very early stage lesions are established (Fig. 7a).

As previously observed, the majority of lesions across all animals (regardless of treatment) were small indolent lesions (93% less than 1 mm^2^), with only a minority rapidly progressing to a very large size (3.5% at or greater than 10 mm^2^) (Fig. 7b). Anti-IL-17 treated animals exhibited a decrease in the number of aggressive lesions >1 mm^2^ (Fig. 7c, d), associated with decreased circulating neutrophils (Fig. 7e), suggesting that anti-IL-17 treatment inhibited lesion progression. A commensurate increase in small lesions (Fig. 7c) suggests that IL-17 blockade did not prevent small lesions from forming but limited their subsequent progression. Finally, when compared with a population of animals identically treated with isotype control (pooled from multiple cohorts), animals treated with anti-IL-17 showed delayed latency to a palpable tumor (Fig. 7f). These results are consistent with a pro-tumor role of IL-17 and possibly neutrophils in aggressive niches, as has been described for N2 neutrophils and/or granulocytic myeloid-derived suppressor cells (G-MDSCs)^50,51^.

To validate the role of pro-tumor neutrophils/G-MDSCs in our model, we next measured the effect of treating lesion-bearing animals with SX-682, a small molecule inhibitor of CXCR1/2 reported to target G-MDSCs (currently in phase I trials for melanoma, NCT03161431). After allowing lesions to establish for 1 week, we switched experimental mice to feed containing SX-682 or kept control mice on standard chow (Fig. 7g). Again, the majority of lesions across all animals were small (97%), with rapidly progressing lesions in minority (<1%, Fig. 7h). Compared to control animals, SX-682-treated animals harbored fewer lesions overall (Fig. 7i), as well as fewer aggressive lesions (Fig. 7i, j), again associated with decreased circulating neutrophils (Fig. 7l). SX-682-treated animals, when compared against a population of animals identically treated with standard chow, showed delayed latency to a palpable tumor (Fig. 7k). Taken together, the decreased frequency of lesion advancement in animals treated with either anti-IL-17 or SX-682 suggests that IL-17 and neutrophils/G-MDSCs were capable of promoting aggressive niche formation and lesion progression.

## Discussion

**Fig. 8:**
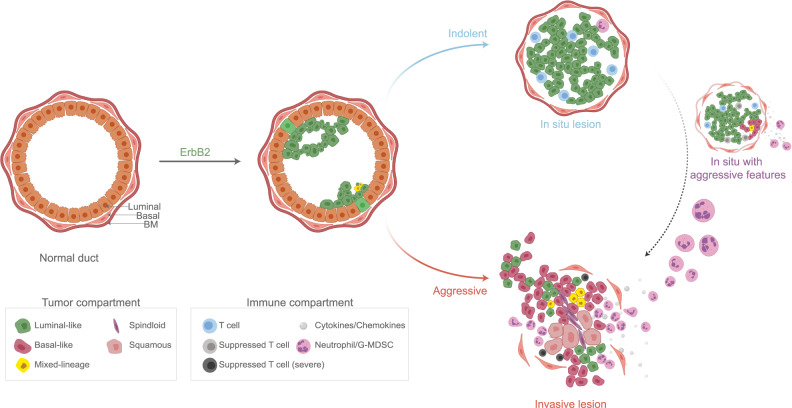
Indolent and aggressive lesions exhibit divergent tumor-immune niches. Following introduction of the caErbB2 oncogene, transformed mammary epithelial cells form very small, early-stage lesions within mammary ducts. Over time, lesions diverge to form either indolent lesions or aggressive lesions. Indolent lesions are characterized by stereotypic, luminal-like populations, and immune infiltration into indolent lesions is limited and tend to be non-suppressed lymphoid (T cells) rather than myeloid. In contrast, aggressive lesions exhibit heterogeneous and functionally divergent tumor populations, including expanded basal-like populations as well as metaplastic and mixed-lineage tumor cells. Immune infiltration into an IL-17-active niche is heavy, primarily comprised of neutrophils or granulocytic myeloid-derived suppressor cells (G-MDSCs), while T cells are diminished in both number and activity. Indolent lesions may transition to become aggressive, evidenced by intermediate lesions that appear indolent but exhibit sub-niche expansion of basal-like cells and recruit granulocytes that are normally restricted to aggressive lesions. BM basement membrane. See also Supplementary Fig. 8.

In this study, we utilized a mouse model of breast cancer that generated both indolent and aggressive lesions to gain insight on features that distinguish these lesion types and to identify potential determinants of lesion aggressiveness. We showed that indolent and aggressive lesions were comprised of distinct tumor-immune cell niches (Fig. 8). Importantly, these distinct niches existed within the same mammary gland, strongly supporting the idea that niche features can be locally determined by spatially restricted collaborative cues that modulate or override systemic conditions and, in this model, a common tumor-initiating event (expression of caErbb2).

We demonstrated that lesions harbor a heterogeneous population of tumor cells that shift in frequency in aggressive lesions relative to indolent lesions. Further, we inferred that luminal-like and basal-like populations within lesions adopt divergent biological roles that become more extreme during disease progression, gaining insight into the functional specialization that accompanies increased cellular, spatial, and resource heterogeneity within the tumor niche^52^. Such insight has been challenging to gain from genomic and transcriptomic analyses that have identified high concordance detected between invasive and non-invasive lesions, but resonate with observations of ecological niche partitioning and division of labor^53^.

We found that aggressive lesions harbored mixed-lineage tumor cells. The emergence of these cells is consistent with similar mixed-lineage populations detected in recent single-cell studies of normal and transformed mammary tissues^54–61^, and suggest that aggressive progression in this model is accompanied by increased tumor cell plasticity. In particular, our observations agree with previous findings that luminal-like cells acquire basal-like features following oncogenic or homeostatic disruption^62–68^. Conversely, we cannot rule out the possibility that the LB population arises from basal-like cells, consistent with basal-compartment cells giving rise to luminal cells in proposed hierarches of normal mammary gland development^69,70^, and with studies showing that adult committed basal cells can be reprogrammed to acquire more luminal-like states^67,71,72^. While our preliminary data inferring copy-number variations from scRNA-seq data did suggest more chromosomal abnormalities in the Basal 1 population compared to all other clusters, further studies are warranted to prove a clonal relationship between Luminal, LB, and Basal cells at the genetic level.

In addition, LB cells may exhibit progenitor-like properties, as suggested by findings that epithelial-to-mesenchymal programs can generate cells with stem-like properties^36^. Such hybrid cells have been shown to promote aggressive behavior, by enhancing tumor initiation, invasion, metastasis, intratumoral heterogeneity, and therapy-resistance^73–77^. These studies and our work highlight the need to define and functionalize various cell states and lineages partaking in breast tumor progression, including those identified in our study. To this end, future work aims to more fully characterize these hybrid mixed-lineage cells in our model and measure their biological potential.

Despite vast tumor cell-intrinsic differences between indolent and aggressive lesions, we found that cells derived from these distinct lesions were functionally indistinguishable from one another when placed in matched microenvironments. These results support a model of conditional lesion aggressiveness in which crosstalk between tumor cells and their local niche play a more critical role in driving aggressive behavior than do major cell-intrinsic transcriptional and phenotypic differences between indolent and aggressive tumor cells^78^. Our observations are in agreement with previous reports that human in situ lesions may harbor occult cells capable of aggressive behavior and invasive growth, and that genetically similar lesions may nonetheless differ in local aggressiveness (in situ vs. invasive)^17,22,79–82^. Thus, this study underscores the ongoing need to understand the role of the tumor niche in driving lesion behavior even in the early stages of breast cancer progression, and to determine how re-shaping the local niche might prevent or control aggressive tumor behavior.

To this end, we described a shift in immune composition between indolent and aggressive lesions, in agreement with previous reports^19,20^. Notably, we observed that aggressive lesions exhibited a lymphoid-to-myeloid switch consistent with findings that tumors recruit myeloid cells that can suppress anti-tumor lymphocytes^83–88^, and that tumor aggressiveness, including the transition from in situ to invasive disease, is modulated by the ability of a lesion niche to dampen anti-tumor immunity^19,48^.

In particular, we identified that S100A8+ neutrophils specifically infiltrated aggressive niches, which also displayed increased IL-17 signaling. Tumor-associated neutrophils, or TANs, have been reported to play dual roles in the tumor microenvironment, including in breast cancer^51,89–91^. The functional role of TANs in DCIS, which have been shown to increase compared to normal tissue, is not known, but may be similarly double-sided^19,92^. In addition, IL-17 signaling may impact tumor cells, for example by regulating tumor cell plasticity, as has been shown in pancreatic lesions^93^. Recent studies have found that IL-17 and TANs can promote the progression of tumors derived from transplanted metastatic or chemoresistant cells^94,95^. In agreement with these findings, in a proof-of-principle study using our in vivo model of spontaneous tumor initiation, we determined that IL-17 and/or TANs played a pro-tumor role even at early stages of aggressive progression, when lesions become locally invasive. Notably, TANs were very tightly associated with the establishment of the aggressive niche such that we could not dissociate these two niche features even with the blockade of neutrophil recruitment. Altogether, our work and others suggest that IL-17 signaling and neutrophil recruitment promote tumor aggressiveness as early as the onset of local invasion, through to metastasis and development of chemoresistance, indicating a large therapeutic window during which targeting IL-17/TANs may yield clinical benefit. Thus, our work warrants further mechanistic investigation of IL-17 signaling and TANs to identify or therapeutically target DCIS lesions that may be capable of aggressive behavior, including the use of SX-682 in patients at risk for aggressive progression. Importantly, since inhibiting neutrophil recruitment reduced but did not eliminate aggressive lesions, it will be essential to account for the likely contributions of additional niche collaborators, including both cellular and acellular elements, such as altered matrix structure and composition^93,96–99^.

Finally, although our findings do not rule out the possibility that indolent and aggressive lesions follow independent natural histories, our observations suggest a putative biological continuum between some indolent and aggressive lesions based on the identification of small lesions bearing aggressive niche features. Importantly, these features may also herald aggressiveness in human lesions that might be otherwise considered indolent. Ongoing work includes profiling human breast lesions in various stages of progression to determine whether and at which point such features arise, to explore their potential clinical utility as predictive markers to strengthen current risk assessment approaches^100^, and to identify whether the populations they mark could serve as therapeutic targets to prevent or delay disease progression. Given that differential activation of oncogene regulatory pathways (including those that define intrinsic breast cancer subtype) could potentially influence downstream immune cell recruitment^101^, our model potentially points to differences in the tumor-immune niches not just between indolent and aggressive lesions but also between different Luminal and Basal subtypes of human breast cancer. Because our model also histologically resembles aggressive metaplastic breast cancer that in rare cases also expresses HER2^102^, similar studies are required in additional models to determine the relationship between indolent and aggressive lesion niche collaborators across a broader spectrum of breast cancer subtypes, and to identify key potential niche targets for treatment stratification and therapeutic intervention.

## Methods

### Animals

Animal work described was performed in accordance with recommendations in the Guide for Care and Use of Laboratory Animals from the National Institutes of Health (NIH) Institutional Animal Care and Use Committee (IACUC) and the American Association for Accreditation of Laboratory Animal Care (AAALAC). Studies were approved by the University of Texas MD Anderson Cancer Center IACUC and, where applicable, the US Army Medical Research and Development Command Animal Care and Use Review Office. Animals were obtained from Jackson Laboratories (*Mus musculus*, FVB/NJ, 001800) for direct use in studies as well as establishment of in-house colonies, and all housing and work carried out in an AAALAC International-accredited and Public Health Service Animal Welfare-assured facility. All animals were euthanized in accordance with NIH and AAALAC guidelines. Animals were housed at 72 °F, 50% humidity, with a 12 h dark/light cycle (6 a.m./6 p.m.).

### Lentivirus production and titering

Constitutively activated ERBB2 (caErbB2) was delivered using a lentiviral vector (FUCGW)^26^. Virus was produced in-house^24,26^ or prepared by the University of Michigan Medical School Biomedical Research Core Facilities. For in-house preparation, lentiviral expression and packaging plasmids (pMDLg/pRRE, pRSV-Rev, VSVg) were transfected with TransIT^®^-LT1 (Mirus) or FuGENE^®^ 6 (Promega) into HEK293T cells. Virus-containing media was filtered through 0.45 μm membrane then subjected to centrifugation at 70,000 g for 3 h to concentrate. Concentrated virus was titered by limiting dilution transduction of 293T cells, followed by the enumeration of percent fluorescent cells. Aliquots of concentrated virus were stored at −80 °C until use. Work described was approved by the MD Anderson Cancer Center Institutional Biosafety Committee.

### Intraductal injection of virus/cells

Adult female virgin FVB mice were anesthetized using isoflurane (2–4%) by way of inhalation. Following confirmation of anesthesia by gentle toe pinch, a small tip (<3 mm) of the #4 nipple was cut with micro-dissecting surgical scissors to expose the nipple duct. Up to 1 × 10^6^ viral particles or 200,000 cells (~10 µl volume) were injected into the nipple duct using 22- to 34-gauge blunt needles fitted to a Hamilton syringe. Mice were palpated for tumors up to three times weekly until study endpoint. Tumor-free survival analyses were performed using *R* (≥3.1.0) and/or GraphPad Prism 8. Lesion-forming capacity of injected cells was analyzed using ELDA^33^.

### Tissue processing (embedding)

Upon collection of mammary glands, tissues were incubated in 10% formalin at 4 °C ~48–72 h until fixation was achieved, then embedded in paraffin. Formalin-fixed paraffin-embedded (FFPE) tissues were sectioned to 3–5 µm in-house, or by the MD Anderson Research Histology Core Laboratory (RHCL), or the Center for Radiation Oncology Research (CROR) Histology Core.

### Tissue staining (H&E and Immunostaining)

Hematoxylin and eosin (H&E) staining was performed by the RHCL or CROR Histology Core. H&E stained slides were scanned at MD Anderson RHCL using the Aperio AT2 slide scanner (Leica Biosystems), and lesions digitally quantified using Aperio ImageScope (Leica Biosystems) or ImageJ^103^ and statistically analyzed using R (≥3.1.0) and/or GraphPad Prism 8. To perform immunostaining, FFPE tissue slides were baked at 65 °C for an hour, and then dewaxed and rehydrated by graded washes in xylene to ethanol. Heat-mediated epitope retrieval was performed by incubating slides in Reveal Decloaker or Nuclear Decloaker solutions (Biocare Medical), heated to 97 °C for 15 min using an EZ-Retriever microwave (BioGenex). Following phosphate-buffered saline (PBS) wash, endogenous peroxidase activity was quenched by incubation of tissues in Dual Endogenous Enzyme Block (Dako) for 10 min at room temperature. Tissues were blocked using Protein Block (Dako) and/or normal serum (Vector ImmPRESS). Slides were incubated with primary antibodies overnight at 4 °C, washed in PBS, then incubated with secondary antibodies. For immunofluorescence, slides were mounted using ProLong™ Gold Antifade Mountant with DAPI (Thermo Fisher). For immunohistochemistry (IHC), slides were incubated with horse radish peroxidase substrate (HRP, Vector ImmPACT) to develop stain, counterstained with hematoxylin QS (Vector), dehydrated through graded ethanol-to-xylene washes, and then mounted using permanent mounting medium (VectraMount). For multiplex IF, slides were prepared using the Opal 7-color Manual IHC Kit per manufacturer’s instructions (Akoya Biosciences). Briefly, following incubation with HRP, slides were stained with tyramide-bound fluorophore, and then treated with additional rounds of HIER, re-staining until finally counterstained with DAPI and mounted with ProLong™ Gold Antifade Mountant with DAPI (Thermo Fisher). Slides were imaged using the Vectra^®^ 3 (Akoya Biosciences) or Vectra^®^ Polaris^TM^ Quantitative Pathology Imaging System (Akoya Biosciences) housed at the MD Anderson Flow Cytometry and Cellular Imaging Core Facility (FCCICF). Slides stained for imaging mass cytometry^104^ were prepared as above for IHC up to incubation with a freshly-prepared cocktail of metal-conjugated primary antibodies, after which slides were washed in PBS, counterstained with 0.625 µM iridium in 1.6% paraformaldehyde for 30 min at room temperature and then air dried. Tissue sections stained with metal-conjugated antibodies were scanned using the Hyperion Imaging System (Fluidigm) housed at the FCCICF, and image data visualized and processed using MCD Viewer (Fluidigm). Images were processed and compiled using Adobe Photoshop and Adobe Illustrator.

The following antibodies were used for immunostaining with IHC, IF, and multiplex IF: CD19 (Thermo Fisher 14-0194-82, 1:500); CD3 (Abcam ab5690, 1:500); CD45 (e-Biosciences 14-0451-85, 1:500); Col IV (Abcam ab6586, 1:750); E-cadherin (CST 3195, 1:500); ER-alpha (Millipore 06-935, 1:500); F4/80 (Thermo MA1-91124, 1:200); HA (BioLegend 901502, 1:500); Ki67 (Abcam ab15580, 1:500); MPO (Abcam ab208670, 1:1000); S100A8 (R&D Systems MAB3059, 1:1000). The following antibodies were used for imaging mass cytometry: CD163 (Abcam ab213612), conjugated to 168 Er, 20 µg/ml; CK5 (Abcam ab214586), 145 Nd, 5–10 µg/ml; CK8 (Abcam ab217173), 154 Sm, 20 µg/ml; Col-IV (Abcam ab6586), 155 Gd, 20 µg/ml; pS6 (CST 4858), 167 Er, 10 µg/ml; S100A8 (R&D MAB3059), 115 In, 5 µg/ml; Vimentin (Abcam 193555), 151 Eu, 10 µg/ml; αSMA (CST 19245), 158 Gd, 10 µg/ml.

### Antibody conjugation

Antibody conjugations were performed at the FCCICF using Maxpar X8 Antibody Labeling reagents (Fludigm) according to the manufacturer’s instructions and as previously described^105^. Briefly, lanthanide metal was incubated with X8 polymer at 37 °C for 40 min, and then washed through a 3 kDa spin column to remove free metal. Commercially obtained carrier-free antibodies (100–200 µg) were washed in R buffer (Fluidigm) using a 50 kDa spin column (EMD Millipore), and then prepared for conjugation through partial reduction of antibody sulfide bonds in 4 mM TCEP (Sigma) in R buffer (37 °C, 30 min). Metal-loaded polymer was combined with reduced antibodies in C-buffer and incubated at 37 °C for 90 min in a water bath. Antibody-metal conjugates were washed four times in W-buffer, quantified by Nanodrop, recovered to 0.5 mg/ml in antibody stabilizer (Candor Biosciences), and stored at 4 °C.

### Tissue processing (digestion)

Resected mammary glands bearing caErbB2-GFP lesions were visualized under a fluorescence stereoscope, and regions harboring aggressive (>2 mm, comprised of invasive lesions) and indolent (<2 mm, enriched for in situ lesions) lesions were dissected away from each other. Tissues were mechanically minced, and then digested to organoids in epithelial cell media [advanced DMEM/F12 (Gibco) with 5% fetal bovine serum (Hyclone), and 1% antibiotic-antimycotic (Corning)], supplemented with 3 mg/ml collagenase (Roche), 0.6 mg/ml hyaluronidase (Sigma), 1.3% bovine serum albumin (Sigma). Tissue digests were agitated by angled rotation for 2–5 h at 37 °C, pelleted by centrifugation at 450 g, and then resuspended in red cell lysis buffer (Sigma). Following wash with epithelial cell media and PBS, pelleted organoids were incubated in 0.25% Trypsin-EDTA (Corning) at room temperature for 5 min. Cells were again washed, pelleted, and resuspended in epithelial cell media supplemented with up to 10U dispase (Stem Cell) and 5 µg DNAase I (Stem Cell), filtered serially through 100 and 70 µm cell strainers (Falcon), and enumerated by trypan blue staining.

### Preparation of isolated cells for injection

Following digestion of tissues down to single cells, samples were stored at 4 °C overnight in Hypothermosol (BioLife Solutions). The following day, tumor cells were washed and resuspended in PBS with 0.5% EDTA, and then isolated by fluorescence-activated cell sorting (FACS) for GFP. Gating was performed using BD FACSDiva 8. Sorted cells were washed with HBSS supplemented with 5% FBS, 10 µM ROCK inhibitor (Sigma), and 500 µm N-acetylcysteine amide (Sigma). For transplantation studies, cells were resuspended in HBSS only and kept on ice until same-day injection.

### Bulk RNA sequencing

For bulk transcriptomic profiling, digested tumor cells were isolated by FACS as above, homogenized using a Bullet Blender (Next Advance) and RNA extracted (Qiagen RNeasy Kit). RNA was treated with DNase (Turbo DNA-Free Kit), and then cleaned and concentrated (Zymo RNA Cleanup Kit), per manufacturers’ instructions. RNA library preparation (NEBNext^®^ Ultra™ RNA Library Prep Kit for Illumina, NEBNext^®^ Poly(A) mRNA Magnetic Isolation Module) and sequencing (Illumina HiSeqX Ten, 150 nucleotide paired-end reads) were performed by Admera Health (South Plainfield, NJ). STAR (v2.6.0b)^106^ was used to align reads to the GRCm38.p6 reference genome. Samtools (v1.8)^107^ was used to sort, convert between formats, and calculate mapping statistics. FastQC (v0.11.5)^108^ was used to check for qualities of the FASTQ reads. Gene annotation was carried out using the GENCODE M19 (Ensembl 94) annotation, which was downloaded from the GENCODE project^109^. Aligned reads were summarized at the gene level by STAR (v2.6.0b)^106^. R (3.6.0) and Bioconductor package DESeq2^110^ was used to identify differentially expressed genes. Read count was first pre-filtered to remove extremely low expressed genes. DESeq2 then carried out read count filtering, normalization, dispersion estimation, and identification of DE. DESeq2 modeled the counts using a negative binomial distribution, followed by the Wald test. The final *p* value was adjusted using the Benjamini & Hochberg method. Heatmaps of unsupervised hierarchical clustering of differentially expressed genes in all samples were plotted. The genes were median-centered for contrast. The clustering was done using Pearson distance and Ward linkage. The Molecular Signatures Database (MSigDB, https://www.gsea-msigdb.org/gsea/msigdb/index.jsp) v7.0 Hallmark gene sets and curated gene sets (C2) were used in GSEA (v4.0.3). The PAM50 subtype predictions were carried out using Bioconductor package genefu (v2.20.0).

### Single-cell RNA sequencing

For single-cell transcriptomic profiling, tumor and immune cells were isolated by FACS for GFP and CD45 (stained using PE anti-mouse CD45 antibody, BioLegend 103106, 0.25 µg per 10^6^ cells in 100 µl). Samples were transferred in media suspension to the MD Anderson Advanced Technology Genomics Core (ATGC, CA016672), where sample concentration and viability were evaluated using automated cell counting (Countess II FL Auto counter), prior to 3′ capture and library preparation (5000 cells/sample, Chromium Single Cell 3′ v2) using the Chromium Controller (10X Genomics) and sequencing (Illumina NextSeq500). Raw FASTQ reads were mapped to mouse mm10 genome by Cell Ranger v3.0.2 to generate the cell-gene count matrix^111^. The matrix file was analyzed by R package Seurat v3.1.0^112^. Specifically, cells with less than 500 UMI count or more than 10% mitochondrial gene UMI count were treated as low-quality cells and excluded from further analysis. Cell cycle scores (S and G2M) were calculated using the gene lists provided by Seurat. The count matrix was then log normalized and multiplied by the scale factor 10,000. Variance-stabilizing transformation method was used to choose the top 2000 variable genes. The package DoubletFinder was used to exclude cells likely to be doublets^113^. A doublet percentage of 5% was used for the estimation. The cells from the same FACS strategy (GFP or CD45) were integrated with Canonical Correlation Analysis and dimension of 30. The integrated data matrices were further scaled and centered, and the effects of UMI counts, mitochondrial RNA proportion, cell cycle scores were also regressed out during this step. The data were further processed and visualized by PCA and Uniform Manifold Approximation and Projection. Smart local moving algorithm was used for cluster identification. DE analysis between groups in each cluster was performed using FindMarkers function. GSVA scores were calculated by R package GSVA v1.36.2^114^. The Hallmarks and KEGG gene lists were downloaded from MSigDB v7.0. Pseudotime analysis was performed by Monocle v2^115^. T cell exhaustion scores were calculated by Seurat AddModuleScore function and T cell exhaustion gene lists from a previous study^116^. For cell ploidy inference, ploidies of single cells were inferred from scRNA-seq data by InferCNV v1.3.3 (https://github.com/broadinstitute/infercnv). Raw count matrix and labels of cells with epithelial lineage (Basal, LB, Luminal 1, and Luminal 2) were input into the software and run with default settings, according to the software manual. Aneuploidy was defined as greater than 70% of the chromosome having inferred abnormalities. Mann–Whitney U tests were performed to compare the differences in aneuploidy.

### In vivo antibody/diet studies

Lentiviral particles were injected intraductally as described above, and lesions were allowed to establish for 1 week, after which treatment commenced. For IL-17 blockade studies, 200 µg each of anti-IL-17 and anti-IL-17R antibodies (Amgen) (or polyclonal IgG control, BioXcell) in PBS were administered intraperitoneally twice per week until study endpoint. For SX-682 studies, experimental animals were placed on medicated diet (Syntrix Pharmaceuticals) formulated with 0.756 grams of SX-682 per kilogram of feed until study endpoint. Food consumption was measured twice weekly to calculate approximate mean daily dose (~80 mg/kg). For both studies, mammary glands were palpated twice weekly for tumors and animals were monitored for potential adverse effects including body weight loss. When the most rapidly growing tumors reached maximum allowable burden, mammary tissues from all animals were collected (to enable evaluation of both tumor latency and lesion burden) and then FFPE as described above.

### Reporting summary

Further information on research design is available in the Nature Research Reporting Summary linked to this article.

## Supplementary information

Supplementary Information

Description of Additional Supplementary Files

Supplementary Data 1

Supplementary Software 1

Reporting Summary

## Data Availability

Source data are provided with this paper. The bulk and single-cell RNA sequencing data generated in this study have been deposited in the Gene Expression Omnibus database under accession codes GSE162005 and GSE161983. Publicly availably MSigDB v7.0 Hallmark, C2 curated (c2), and CP:KEGG gene sets can be accessed at https://www.gsea-msigdb.org/gsea/msigdb/index.jsp. The remaining data are available within the Article, Supplementary Information or Source Data file. Source data are provided with this paper.

## Source data

Source Data

## Code availability

An R package ‘Scillus’ was used for plotting the scRNA-seq datasets; Scillus is a Seurat and ggplot2 wrapper for providing enhanced data visualization functionalities and code can be found at https://github.com/xmc811/Scillus and https://github.com/xmc811/ScRNA-Seq_HER2. The corresponding DOIs are as follows: Scillus; 10.5281/zenodo.5138771 and ScRNA-Seq_HER2; 10.5281/zenodo.5129249. Code used to analyze bulk RNAseq data is provided with this publication as Supplementary Software 1.
