# Supplementary material for: Single-cell evaluation reveals shifts in the tumor-immune niches that shape and maintain aggressive lesions in the breast
Source: Nat Commun. 2021 Aug 18;12:5024. doi: 10.1038/s41467-021-25240-z (PMC8373912; doi:10.1038/s41467-021-25240-z)
Supplement: Supplementary file 1 — Supplementary Information [file 41467_2021_25240_MOESM1_ESM.pdf]

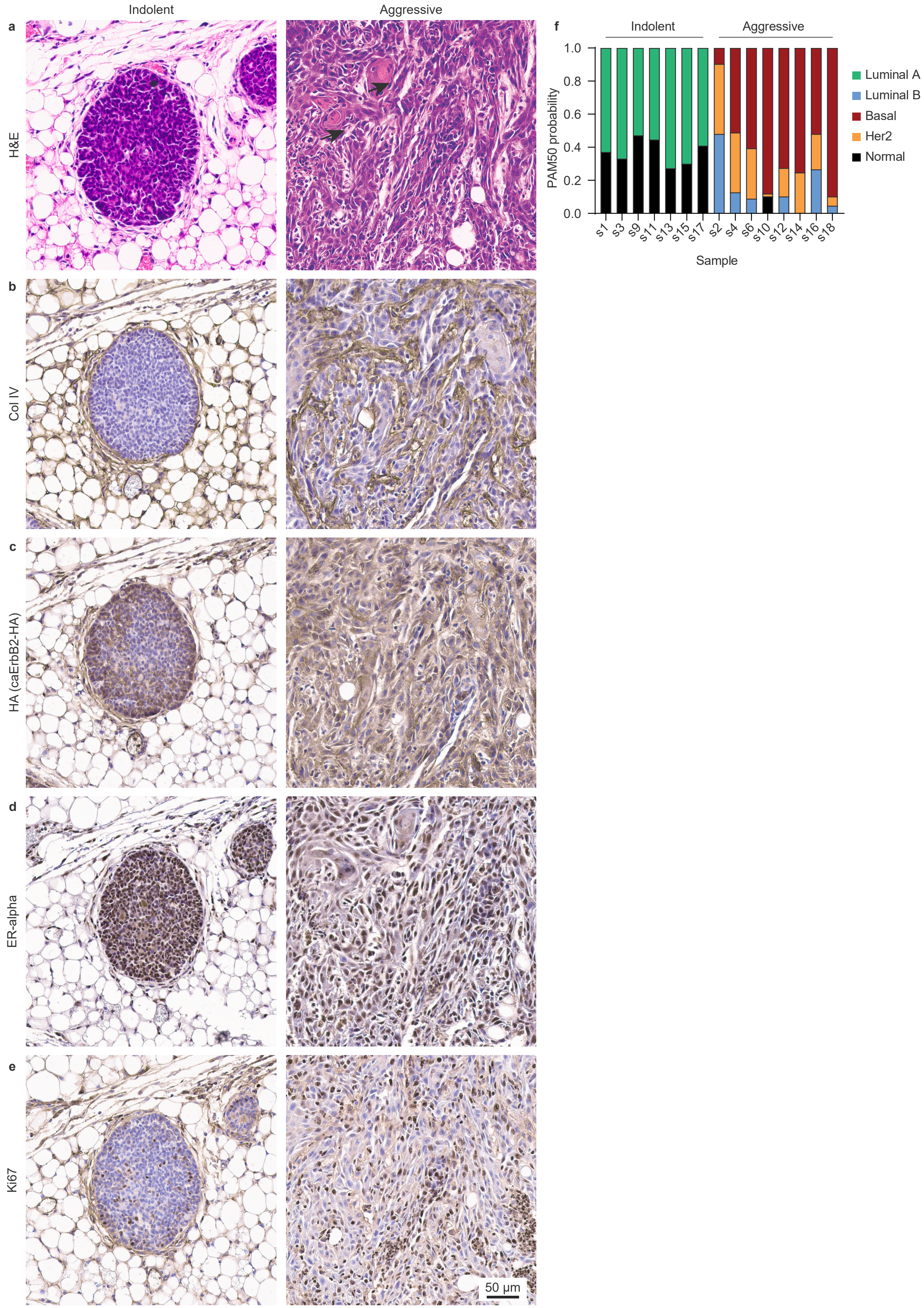
Indolent
Aggressive
a
H&E
b
Col IV
c
HA (caErbB2-HA)
d
ER-alpha
e
Ki67
50 µm
f
Indolent
Aggressive
PAM50 probability
1.0
0.8
0.6
0.4
0.2
0.0
s1
s3
s9
s11
s13
s15
s17
s2
s4
s6
s10
s12
s14
s16
s18
Sample
Luminal A
Luminal B
Basal
Her2
Normal

Supplementary figure 1: Characterization of indolent and aggressive lesions using St. Gallen surrogates and PAM50 intrinsic subtyping

(A-E) Indolent and aggressive lesions were visualized by H&E (A) and immunostained for collagen IV (basement membrane; B), HA (HA-tagged caErbB2 oncogene; C), estrogen receptor alpha (hormone receptor status; D), and Ki67 (proliferation; E). Arrows point to examples of keratin pearl-like structures. Representative images of serial sections shown from n=3 animals.

(F) PAM50 subtype probability of indolent and aggressive samples, calculated from bulk transcriptomic profiles. Each bar represents one sample. Probability score for each intrinsic subtype is shown if greater than zero, for a total probability of 1.0 for each sample.

Source data are provided as a Source Data file.

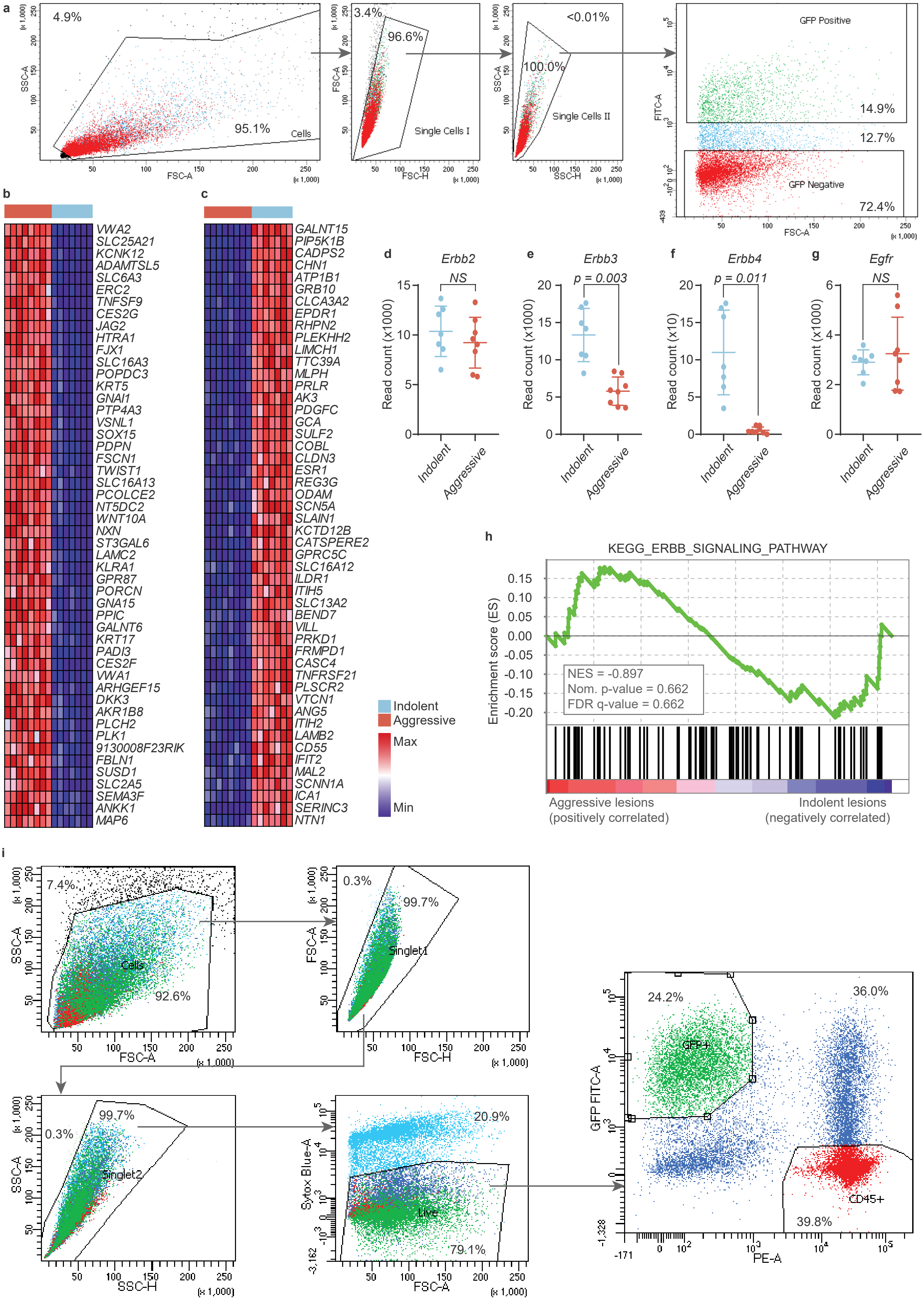
a
4.9%
95.1%
Cells
SSC-A
FSC-A
(x 1,000)
3.4%
96.6%
Single Cells I
FSC-A
FSC-H
<0.01%
100.0%
Single Cells II
SSC-A
SSC-H
GFP Positive
14.9%
12.7%
GFP Negative
72.4%
FITC-A
FSC-A
b
VWA2
SLC25A21
KCNK12
ADAMTSL5
SLC6A3
ERC2
TNFSF9
CES2G
JAG2
HTRA1
FJX1
SLC16A3
POPDC3
KRT5
GNAI1
PTP4A3
VSNL1
SOX15
PDPN
FSCN1
TWIST1
SLC16A13
PCOLCE2
NT5DC2
WNT10A
NXN
ST3GAL6
LAMC2
KLRA1
GPR87
PORCN
GNA15
PPIC
GALNT6
KRT17
PADI3
CES2F
VWA1
ARHGEF15
DKK3
AKR1B8
PLCH2
PLK1
9130008F23RIK
FBLN1
SUSD1
SLC2A5
SEMA3F
ANKK1
MAP6
c
GALNT15
PIP5K1B
CADPS2
CHN1
ATP1B1
GRB10
CLCA3A2
EPDR1
RHPN2
PLEKHH2
LIMCH1
TTC39A
MLPH
PRLR
AK3
PDGFC
GCA
SULF2
COBL
CLDN3
ESR1
REG3G
ODAM
SCN5A
SLAIN1
KCTD12B
CATSPERE2
GPRC5C
SLC16A12
ILDR1
ITIH5
SLC13A2
BEND7
VILL
PRKD1
FRMPD1
CASC4
TNFRSF21
PLSCR2
VTCN1
ANG5
ITIH2
LAMB2
CD55
IFIT2
MAL2
SCNN1A
ICA1
SERINC3
NTN1
Indolent
Aggressive
Max
Min
d
Erbb2
NS
Read count (x1000)
e
Erbb3
p = 0.003
Read count (x1000)
f
Erbb4
p = 0.011
Read count (x10)
g
Egfr
NS
Read count (x1000)
Indolent
Aggressive
h
KEGG_ERBB_SIGNALING_PATHWAY
Enrichment score (ES)
NES = -0.897
Nom. p-value = 0.662
FDR q-value = 0.662
Aggressive lesions
(positively correlated)
Indolent lesions
(negatively correlated)
i
7.4%
Cells
92.6%
SSC-A
FSC-A
0.3%
99.7%
Singlet1
FSC-A
FSC-H
0.3%
99.7%
Singlet2
SSC-A
SSC-H
20.9%
Live
79.1%
Sytox Blue-A
FSC-A
24.2%
GFP+
36.0%
CD45+
39.8%
GFP FITC-A
PE-A

Supplementary figure 2: Transcriptomic analysis of indolent versus aggressive tumor cells

(A) Representative flow cytometry gating strategy to isolate GFP-expressing cells. Arrows indicate parent population for sequential gating. This or similar flow cytometry gating strategy was used to isolate tumor cells for bulk RNAseq and intraductal injection of tumor cells (Figure 2).

(B, C) Top 50 differentially expressed genes in indolent versus aggressive tumor samples. Each column represents one sample. Top 50 genes over-expressed in aggressive lesions are shown in (B), while the 50 most under-expressed are shown in (C).

(D-G) Read counts of *Erbb2* (D) and Erbb family members *Erbb3* (E), *Erbb4* (F), and *Egfr* (G) in indolent and aggressive lesions. P-value was calculated using two-tailed Welch's t-test; center line, mean; error bars, standard deviation (S.D.) Each dot represents one sample.

(H) Gene set enrichment plot for ERBB signaling (KEGG).

(I) Representative flow cytometry gating strategy to isolate GFP- and CD45-expressing cells. Arrows indicate parent population for sequential gating. This flow cytometry gating strategy was used to isolate tumor cells for scRNA-seq (Figures 3, 6).

Source data are provided as a Source Data file.

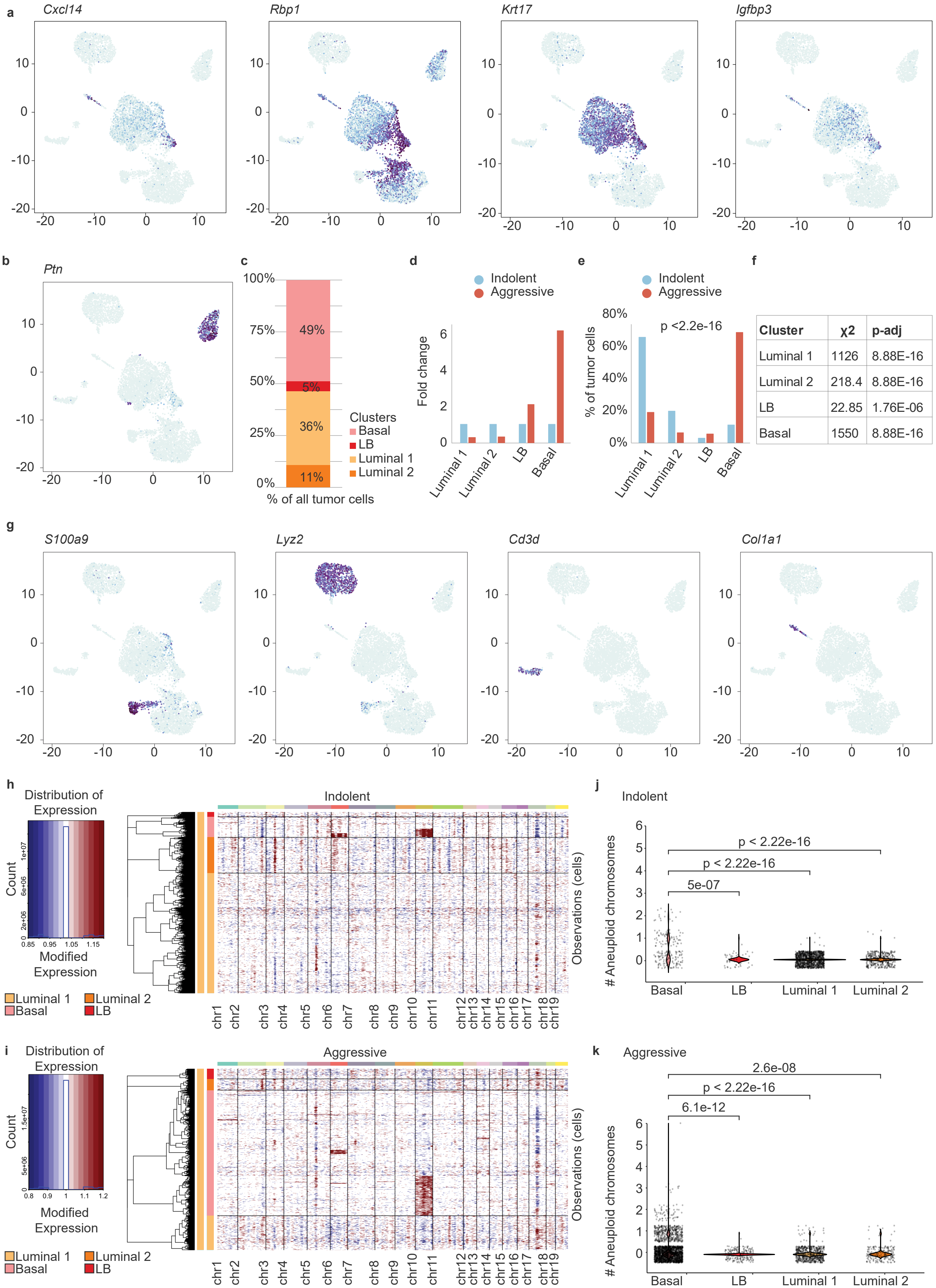

Cluster	χ2	p-adj
Luminal 1	1126	8.88E-16
Luminal 2	218.4	8.88E-16
LB	22.85	1.76E-06
Basal	1550	8.88E-16

Supplementary figure 3: Biomarker expression across tumor subpopulations

(A, B) UMAP plots colored by additional markers identified as differentially expressed in the LB (A) and Luminal 2 (B) tumor populations.

(C) Proportions of tumor clusters from both indolent and aggressive lesions, combined.

(D-F) Bar plot of tumor clusters, with aggressive clusters represented as fold change in proportion relative to corresponding indolent clusters (D), or as a percentage of tumor cells from either indolent or aggressive lesions (E). Chi-square test ($p<0.0001$, $\chi 2 = 1734$, $df = 3$) was used to compared shift in proportions between indolent and aggressive lesions. (F) Proportion of each cluster in indolent and aggressive lesions was compared by Chi-square test, with Holm-Sidak adjusted p-value for each cluster listed.

(G) UMAP plots colored by differentially expressed markers for contaminant populations, including *S100a9* for neutrophils/granulocytes, *Lyz2* for macrophages, *Cd3d* for T cells, and *Col1a1* for fibroblasts.

(H, I) Inferred copy number variation (CNV) of cells from indolent (H) or aggressive (I) lesions, with histogram showing distribution of expression (left) and heatmap of modified expression per cell, organized by chromosome (right).

(J, K) Violin and jittered scatter plot of inferred aneuploidy of cells from indolent (J) and aggressive (K) lesions, by cluster. Each dot represents one cell. Chromosomes exhibiting greater than 70% inferred abnormalities were considered aneuploid. Two-sided Mann–Whitney U test was used to compare groups . (J) W statistics 6652, 164444, 48807, for ‘Basal vs LB’, ‘Basal vs Luminal 1’, and ‘Basal vs Luminal 2’, respectively. (K) W statistics 253371, 835185, 263638, for ‘Basal vs LB’, ‘Basal vs Luminal 1’, and ‘Basal vs Luminal 2’, respectively.

Source data are provided as a Source Data file.

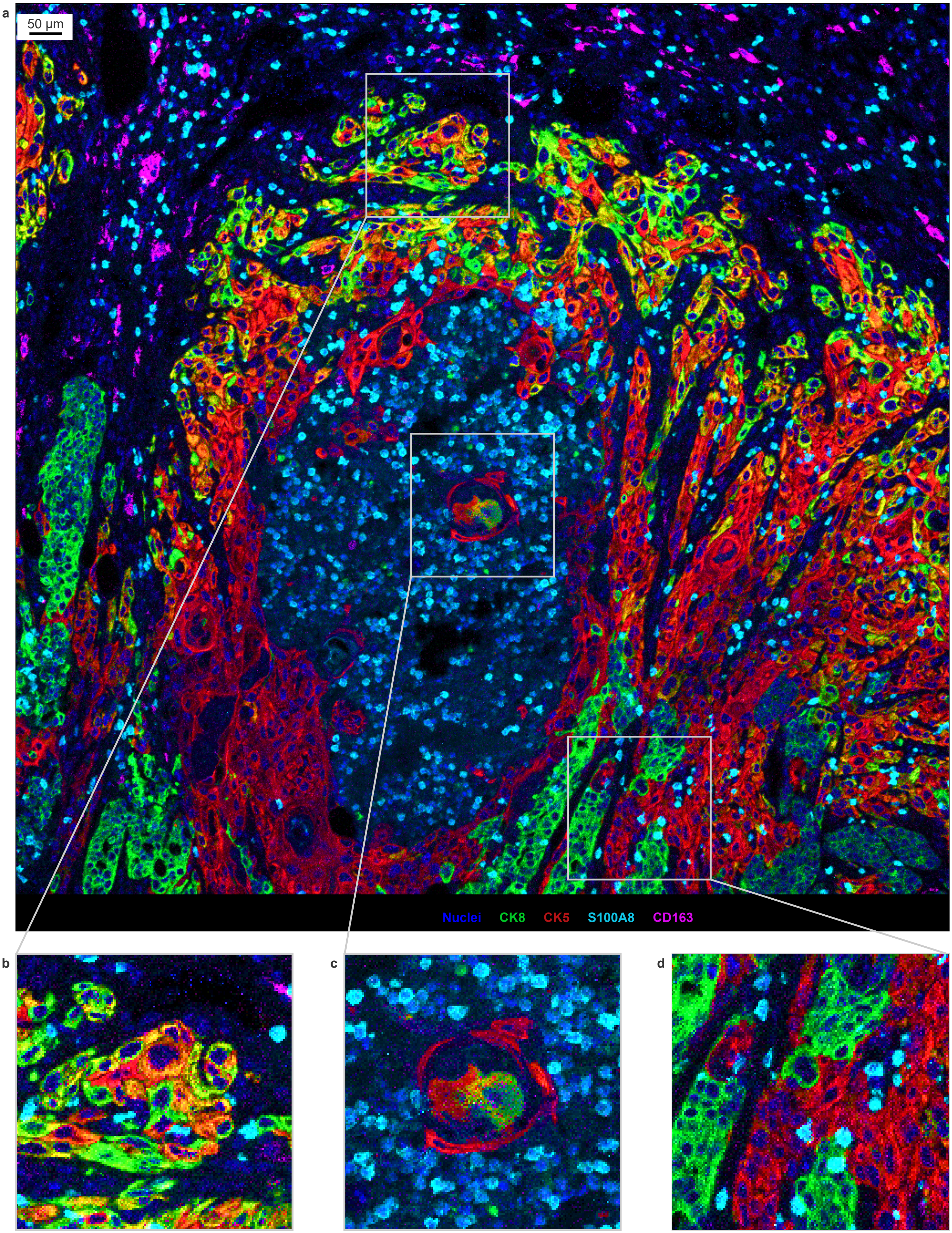
a
50 µm
Nuclei
CK8
CK5
S100A8
CD163
b
c
d

Supplementary figure 4: Multi-parametric imaging reveals spatial intra-lesion heterogeneity of tumor subpopulations

Mammary tissues bearing lesions were stained using a cocktail of antibodies and visualized using imaging mass cytometry. Lesions were stained for CK8 (luminal marker, pseudocolor green), CK5 (basal marker, red), S100A8 (neutrophils/granulocytes, cyan), and CD163 (M2 macrophages, magenta). An area exhibiting a large population of mixed linage tumor cells was selected as an example of a regional metaplasia occasionally observed in aggressive lesions (A). Three regions of interest are highlighted: (B) a sub-lesion niche harboring several adjacent CK8/CK5 double-positive cells; (C) a basal-luminal pair of cells, in which CK5 is detected in the CK8-positive cell along a gradient across the cell body, with the highest expression nearest its closely apposed CK5-positive neighbor; (D) a contrasting sub-lesion niche harboring adjacent clusters of CK8 or CK5 single-positive cells.

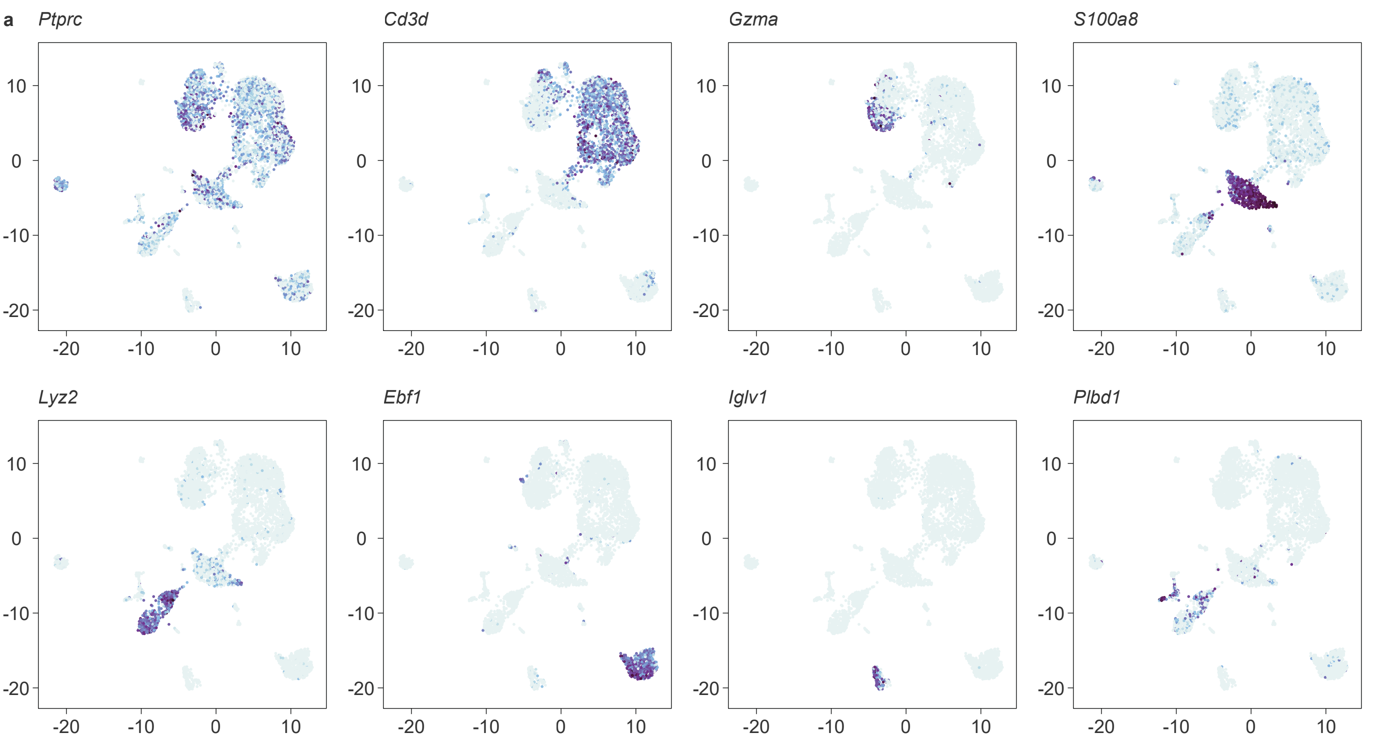

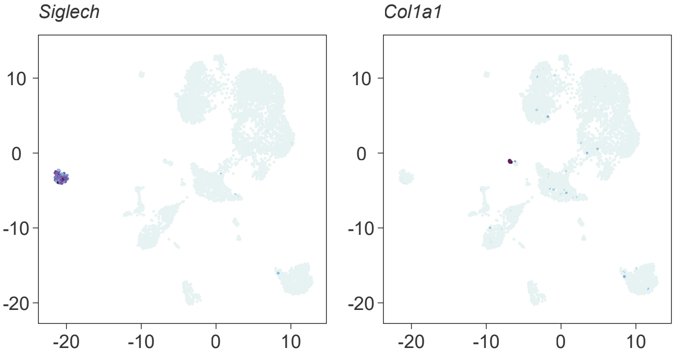

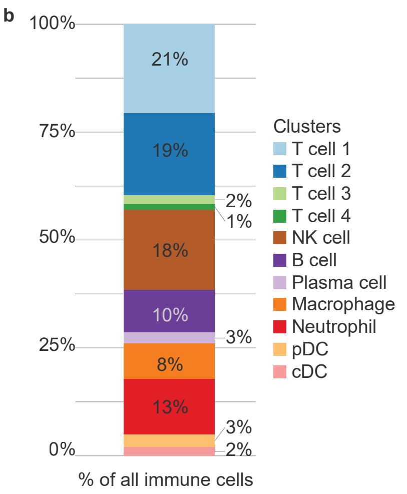

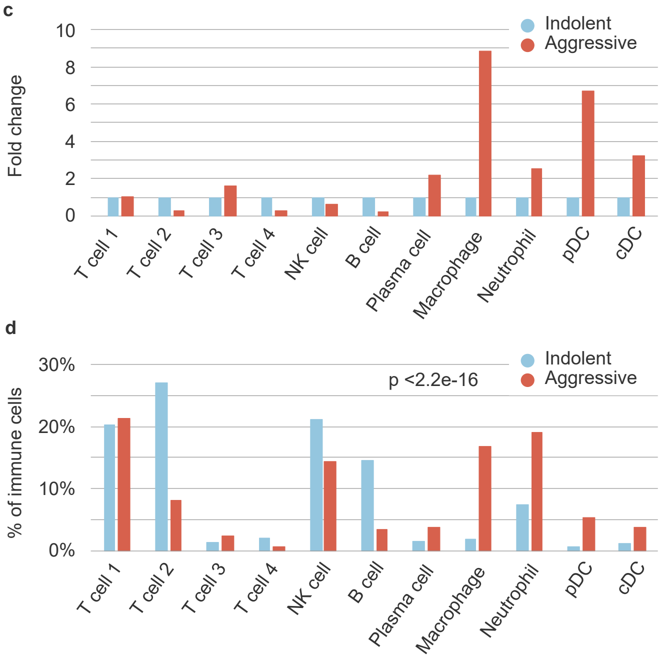

e

Cluster	χ2	p-adj
T cell 1	0.86	0.353
Tcell 2	312.4	2.44E-15
T cell 3	7.004	0.0162
T cell 4	16.97	1.14E-04
NK cell	41.04	7.45E-10
B cell	181.9	2.44E-15
Plasma cell	23.42	5.56E-06
Macrophage	389.5	2.44E-15
Neutrophil	165.5	2.44E-15
pDC	104.1	2.44E-15
cDC	41.57	6.84E-10

Supplementary figure 5: Indolent and aggressive lesions are associated with a diverse immune microenvironment

(A) UMAP plots of all immune cells, colored by expression of *Ptprc* (CD45, all immune cells), *Cd3d* (T cell clusters 1-4), *Gzma* (NK cells), *S100a8* (neutrophils/granulocytes), *Lyz2* (macrophages), *Ebf1* (B cells), *Iglv1* (Plasma cells), *Plbd1* (cDCs), *Siglech* (pDCs), and *Col1a1* (fibroblasts).

(B) Proportions of immune clusters from both indolent and aggressive lesions, combined.

(C-E) Bar plot of immune clusters, with aggressive clusters represented as fold change in proportion relative to corresponding indolent clusters (C), or as a percentage of immune cells from either indolent or aggressive lesions (D). Chi-square test ($p<0.0001$, $\chi2 = 1141$, $df = 10$) was used to compared shift in proportions between indolent and aggressive lesions. (E) Proportion of each cluster in indolent and aggressive lesions was compared by Chi-square test, with Holm-Sidak adjusted p-value for each cluster listed.

Source data are provided as a Source Data file.

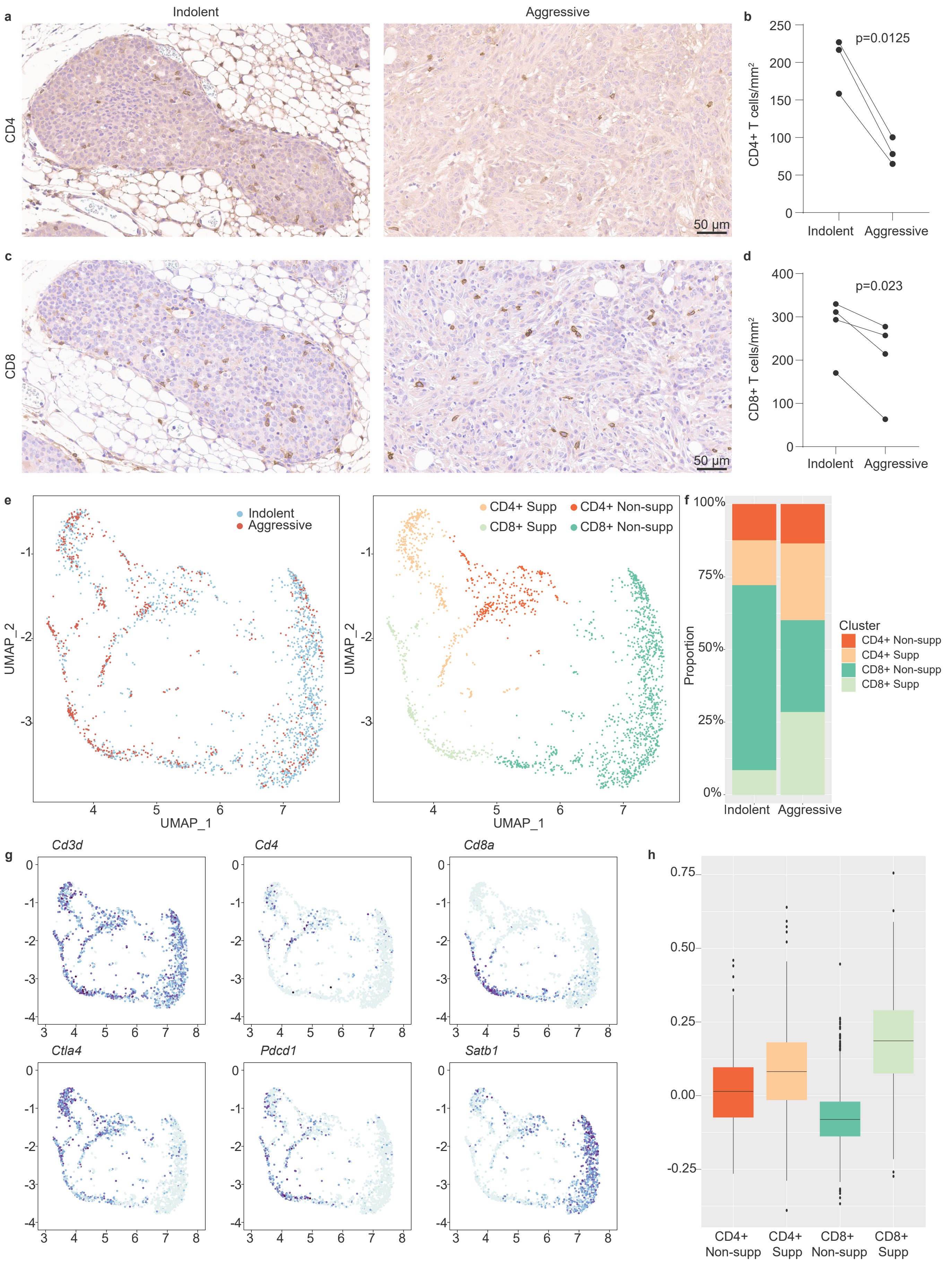

a
Indolent
Aggressive
CD4
50 µm
b
p=0.0125
CD4+ T cells/mm²
Indolent
Aggressive
c
CD8
50 µm
d
p=0.023
CD8+ T cells/mm²
Indolent
Aggressive
e
Indolent
Aggressive
UMAP_1
UMAP_2
CD4+ Supp
CD4+ Non-supp
CD8+ Supp
CD8+ Non-supp
f
Proportion
Indolent
Aggressive
Cluster
CD4+ Non-supp
CD4+ Supp
CD8+ Non-supp
CD8+ Supp
g
Cd3d
Cd4
Cd8a
Ctla4
Pdcd1
Satb1
h
CD4+ Non-supp
CD4+ Supp
CD8+ Non-supp
CD8+ Supp

Supplementary figure 6: The aggressive niche exhibits contracted and suppressed T cell populations

(A-D) Validation and quantification of T cell populations infiltrating indolent and aggressive lesions. T-Helper cells and CTLs were identified by immunohistochemical staining against, respectively CD4 (A, n=3 animals) and CD8 (C, n=4 animals). The numbers of T-helper cells and CTLs per lesion area were quantified (B, D). Ten each of indolent and aggressive lesions per animal were evaluated; data point indicates total positive-staining cells over total lesion area per lesion type per animal. Each pair of linked data points indicates one animal. P-value was calculated by two-sided paired samples t-test.

(E-G) Increased proportion of suppressed CD4+ and CD8+ T cells in aggressive lesions. Cells inferred to be T cells from analysis of the total immune fraction were subset and re-clustered. UMAP plot shows T cells from indolent and aggressive lesions computationally merged (E, left panel) and clustered as four populations (E, right panel). The proportion of each T cell population within indolent and aggressive lesions is shown in (F). Clusters were identified as suppressed or non-suppressed CD4+ and CD8+ cells, based on biomarker expression of *Cd4, Cd8a* for CD4+ and CD8+ populations, *Ctla4* and *Pdcd1* for suppressed populations, and *Satb1* for non-suppressed populations (G) and exhaustion scores calculated from expression of genes associated with immune suppression (H; center line, median; box limits, upper and lower quartiles; whiskers, 1.5x interquartile range; points, outliers). Supp – suppressed; Non-supp – Non-suppressed.

Source data are provided as a Source Data file.

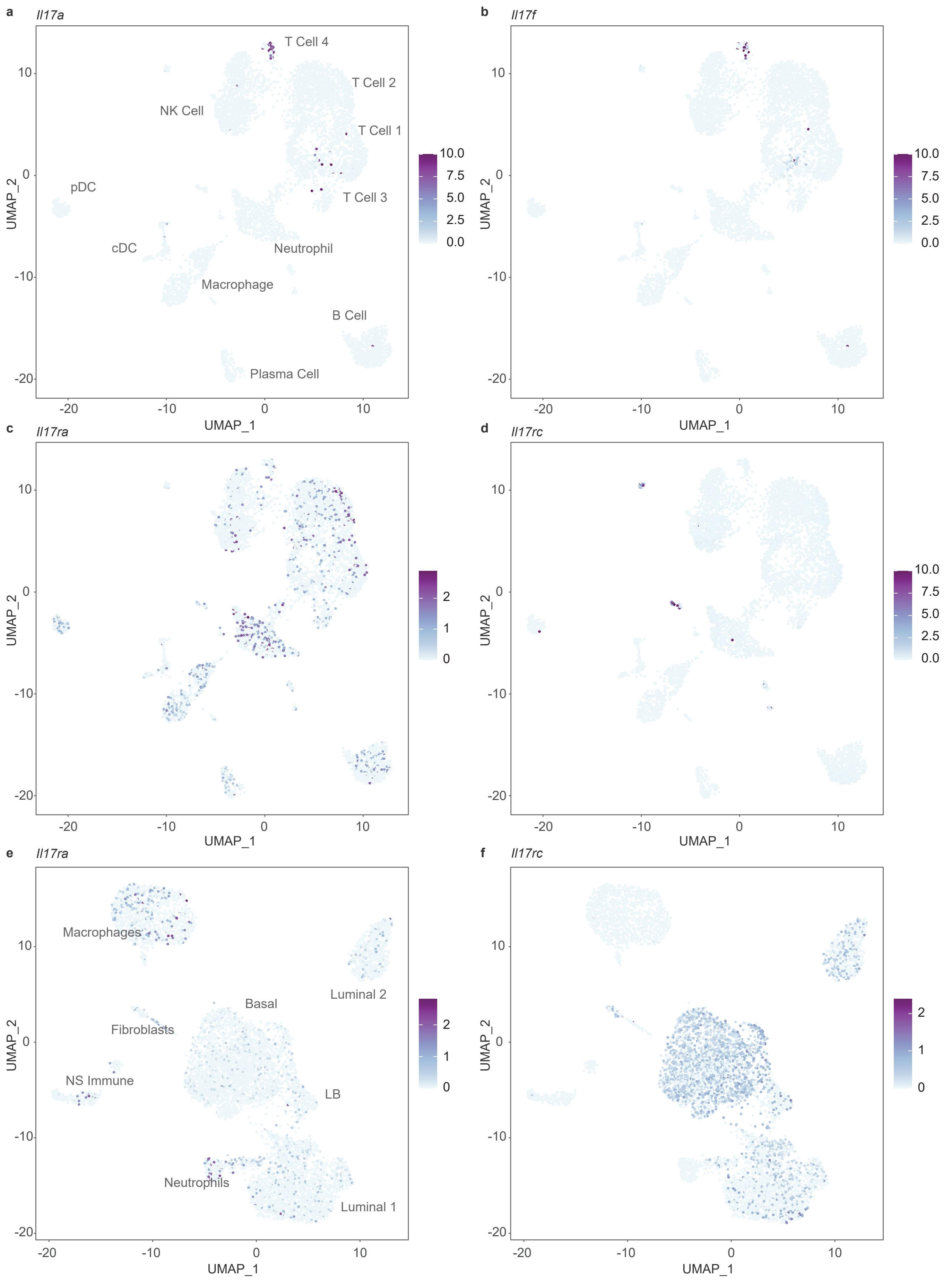
a
Il17a
T Cell 4
T Cell 2
NK Cell
T Cell 1
pDC
T Cell 3
cDC
Neutrophil
Macrophage
B Cell
Plasma Cell
UMAP_1
UMAP_2
b
Il17f
c
Il17ra
d
Il17rc
e
Il17ra
Macrophages
Luminal 2
Basal
Fibroblasts
NS Immune
LB
Neutrophils
Luminal 1
f
Il17rc
10.0
7.5
5.0
2.5
0.0

Supplementary figure 7: Specific populations within the immune niche express IL-17 ligands and receptors

(A, B) Inferred production of IL-17A/F ligands by immune cluster T cell 4. UMAP plots of the immune fraction of lesions (indolent and aggressive lesions computationally merged), colored by expression of *Il17a* (A) and *Il17f* (B).

(C, D) UMAP plots of the immune fractions of lesions (indolent and aggressive lesions computationally merged), colored by expression of *Il17ra (*C) or *Il17rc* (D) to identify populations that potentially respond to IL-17A/F ligands in the niche.

(E, F) UMAP plots of the tumor fractions of lesions (indolent and aggressive lesions computationally merged), colored by expression of *Il17ra* (E) or *Il17rc* (F) to identify populations that potentially respond to IL-17A/F ligands in the niche.

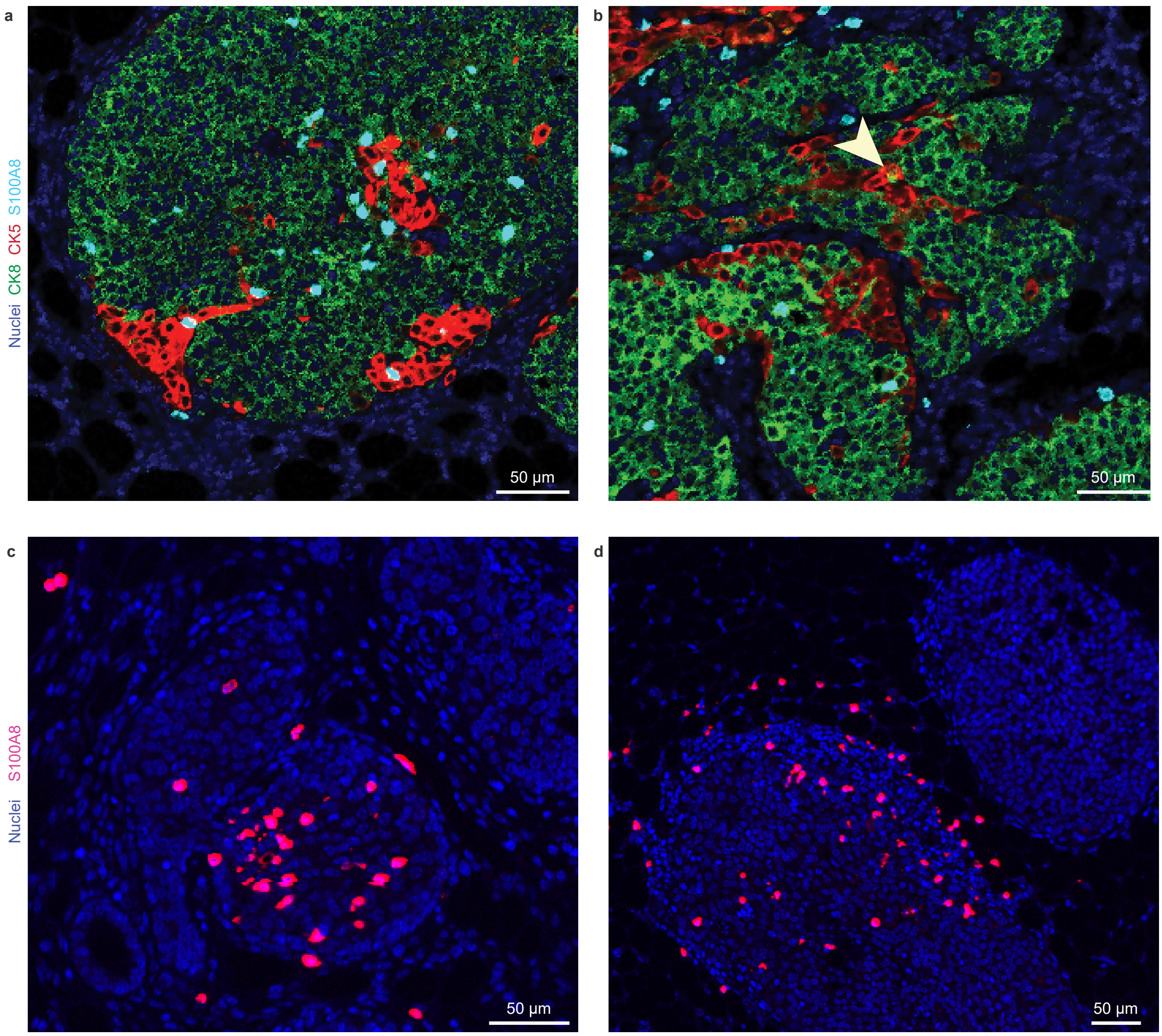

Supplementary figure 8: Mammary glands bear intermediate lesions exhibiting sub-niche expansion of CK5+ cells and recruitment of S100A8+ cells

(A, B) Mammary tissues bearing lesions were stained using a cocktail of antibodies and visualized using imaging mass cytometry. Lesions were stained for CK8 (luminal marker, pseudocolor green), CK5 (basal marker, red), and S100A8 (neutrophils/granulocytes, cyan). Arrow indicates cell expressing both CK5 and CK8 markers.

(C, D) Mammary tissues bearing lesions were stained by immunofluorescence against S100A8 (magenta). Representative images shown from n=7 animals.

Supplementary table 1

NAME	NES	FDR q-val
HALLMARK_E2F_TARGETS	3.3707712	0
HALLMARK_MYC_TARGETS_V1	3.2153695	0
HALLMARK_G2M_CHECKPOINT	3.0851498	0
HALLMARK_MTORC1_SIGNALING	2.4880264	0
HALLMARK_MYC_TARGETS_V2	2.45109	0
HALLMARK_GLYCOLYSIS	2.1354225	0
HALLMARK_P53_PATHWAY	1.9799442	2.11E-04
HALLMARK_EPITHELIAL_MESENCHYMAL_TRANSITION	1.857435	0.001363055
HALLMARK_MITOTIC_SPINDLE	1.84175	0.001211604
HALLMARK_DNA_REPAIR	1.7755426	0.003086696
HALLMARK_CHOLESTEROL_HOMEOSTASIS	1.7321427	0.004025078
HALLMARK_OXIDATIVE_PHOSPHORYLATION	1.7000185	0.004986445
HALLMARK_HYPOXIA	1.6936828	0.004687239
HALLMARK_UNFOLDED_PROTEIN_RESPONSE	1.4833199	0.028203754
HALLMARK_BILE_ACID_METABOLISM	-1.5287917	0.03414649
HALLMARK_INTERFERON_GAMMA_RESPONSE	-1.8879664	0.002147677
HALLMARK_INTERFERON_ALPHA_RESPONSE	-2.1259065	0

Supplementary Table 1: GSEA normalized enrichment scores and FDR q-val. Gene set enrichment analysis (GSEA) results of indolent versus aggressive samples of Hallmarks pathways with FDR <0.05 shown in order of normalized enrichment score (NES). Positive NES indicates enrichment in aggressive samples.

Supplementary table 2

NAME	NES	FDR q-val
KEGG_DNA_REPLICATION	2.745256	0
KEGG_CELL_CYCLE	2.705567	0
KEGG_RIBOSOME	2.637136	0
KEGG_PROTEASOME	2.4070776	0
KEGG_P53_SIGNALING_PATHWAY	2.3494546	0
KEGG_SPLICEOSOME	2.3149905	0
KEGG_MISMATCH_REPAIR	2.2308097	1.72E-04
KEGG_ONE_CARBON_POOL_BY_FOLATE	2.1944852	1.50E-04
KEGG_RNA_DEGRADATION	2.1558433	1.34E-04
KEGG_HOMOLOGOUS_RECOMBINATION	2.1504698	1.20E-04
KEGG_NUCLEOTIDE_EXCISION_REPAIR	2.0695267	4.52E-04
KEGG_PYRIMIDINE_METABOLISM	2.011158	9.19E-04
KEGG_STEROID_BIOSYNTHESIS	1.8167285	0.010482642
KEGG_PARKINSONS_DISEASE	1.8026899	0.010843752
KEGG_HEDGEHOG_SIGNALING_PATHWAY	1.8001201	0.010531586
KEGG_PURINE_METABOLISM	1.796613	0.01015158
KEGG_RNA_POLYMERASE	1.7702446	0.011882625
KEGG_BASE_EXCISION_REPAIR	1.7601753	0.012806135
KEGG_OOCYTE_MEIOSIS	1.6888063	0.025141364
KEGG_CITRATE_CYCLE_TCA_CYCLE	1.645398	0.03692743
KEGG_PROGESTERONE_MEDIATED_OOCYTE_MATURATION	1.6277903	0.04052998
KEGG_ARGININE_AND_PROLINE_METABOLISM	1.614741	0.043765143
KEGG_AXON_GUIDANCE	1.6139402	0.04218062
KEGG_BLADDER_CANCER	1.6074408	0.042514555
KEGG_ALDOSTERONE_REGULATED_SODIUM_REABSORPTION	-1.9231448	0.029644854

Supplementary Table 2: GSEA normalized enrichment scores and FDR q-val. Gene set enrichment analysis (GSEA) results of indolent versus aggressive samples of KEGG pathways with FDR <0.05 shown in order of normalized enrichment score (NES). Positive NES indicates enrichment in aggressive samples.
