# Supplementary material for: Single-cell evaluation reveals shifts in the tumor-immune niches that shape and maintain aggressive lesions in the breast
Source: Nat Commun. 2021 Aug 18;12:5024. doi: 10.1038/s41467-021-25240-z (PMC8373912; doi:10.1038/s41467-021-25240-z)
Supplement: Supplementary file 2 — Description of Additional Supplementary Files [file 41467_2021_25240_MOESM2_ESM.pdf]

Description of Additional Supplementary Files

File Name: Supplementary Data 1
Description: Bulk and single cell RNAseq DE genes

File Name: Supplementary Software 1
Description: Shell scripts, R codes, and example reference and data files for bulk RNASeq data analysis.
