# Supplementary material for: Single-cell evaluation reveals shifts in the tumor-immune niches that shape and maintain aggressive lesions in the breast
Source: Nat Commun. 2021 Aug 18;12:5024. doi: 10.1038/s41467-021-25240-z (PMC8373912; doi:10.1038/s41467-021-25240-z)
Supplement: Supplementary file 5 — Reporting Summary [file 41467_2021_25240_MOESM5_ESM.pdf]

nature research

Corresponding author(s): Helen Piwnica-Worms

Last updated by author(s): Jun 29, 2021

Reporting Summary

Nature Research wishes to improve the reproducibility of the work that we publish. This form provides structure for consistency and transparency in reporting. For further information on Nature Research policies, see our Editorial Policies and the Editorial Policy Checklist.

Statistics

For all statistical analyses, confirm that the following items are present in the figure legend, table legend, main text, or Methods section.

n/a	Confirmed	
☐	☒	The exact sample size (n) for each experimental group/condition, given as a discrete number and unit of measurement
☐	☒	A statement on whether measurements were taken from distinct samples or whether the same sample was measured repeatedly
☐	☒	The statistical test(s) used AND whether they are one- or two-sided<br>*Only common tests should be described solely by name; describe more complex techniques in the Methods section.*
☒	☐	A description of all covariates tested
☐	☒	A description of any assumptions or corrections, such as tests of normality and adjustment for multiple comparisons
☐	☒	A full description of the statistical parameters including central tendency (e.g. means) or other basic estimates (e.g. regression coefficient) AND variation (e.g. standard deviation) or associated estimates of uncertainty (e.g. confidence intervals)
☐	☒	For null hypothesis testing, the test statistic (e.g. F, t, r) with confidence intervals, effect sizes, degrees of freedom and P value noted<br>*Give P values as exact values whenever suitable.*
☒	☐	For Bayesian analysis, information on the choice of priors and Markov chain Monte Carlo settings
☒	☐	For hierarchical and complex designs, identification of the appropriate level for tests and full reporting of outcomes
☒	☐	Estimates of effect sizes (e.g. Cohen's d, Pearson's r), indicating how they were calculated

Our web collection on statistics for biologists contains articles on many of the points above.

Software and code

Policy information about availability of computer code

Data collection	No software was used to collect data
Data analysis	R (>=3.1.0), GraphPad Prism 8, ImageJ 1.52b, BD FACSDiva 8, ELDA (Journal of Immunological Methods 347, 70-78), STAR (v2.6.0b), Samtools (v1.8), FastQC (v0.11.5), Bioconductor package DESeq2, Bioconductor package genefu (v2.20.0), GSEA 4.0.3, Seurat v3.1.0, DoubletFinder, Smart local moving (SLM) algorithm, FindMarkers, GSVA v1.36.2, Monocle v2, Seurat AddModuleScore function, Cell Ranger v3.0.2, InferCNV v1.3.3 (https://github.com/broadinstitute/infercnv).<br>Custom: Scillus 0.4.0 (https://github.com/xmc811/Scillus) and https://github.com/xmc811/ScRNA-Seq_HER2

For manuscripts utilizing custom algorithms or software that are central to the research but not yet described in published literature, software must be made available to editors and reviewers. We strongly encourage code deposition in a community repository (e.g. GitHub). See the Nature Research guidelines for submitting code & software for further information.

Data

Policy information about availability of data

All manuscripts must include a data availability statement. This statement should provide the following information, where applicable:

- Accession codes, unique identifiers, or web links for publicly available datasets
- A list of figures that have associated raw data
- A description of any restrictions on data availability

Source data are provided with this paper. The bulk and single cell RNA sequencing data generated in this study have been deposited in the Gene Expression Omnibus database under accession codes GSE162005 (https://www.ncbi.nlm.nih.gov/geo/query/acc.cgi?acc=GSE162005) and GSE161983 (https://www.ncbi.nlm.nih.gov/geo/query/acc.cgi?acc=GSE161983). MSigDB v7.0 hallmark gene sets and curated gene sets (c2) can be accessed at https://www.gsea-msigdb.org/gsea/msigdb/index.jsp. The remaining data are available within the Article, Supplementary Information or Source Data file.

Field-specific reporting

Please select the one below that is the best fit for your research. If you are not sure, read the appropriate sections before making your selection.

- [x] Life sciences
- [ ] Behavioural & social sciences
- [ ] Ecological, evolutionary & environmental sciences

For a reference copy of the document with all sections, see nature.com/documents/nr-reporting-summary-flat.pdf

Life sciences study design

All studies must disclose on these points even when the disclosure is negative.

Sample size	Initial study designs utilized at least n=3 animals. Animal number was adjusted based on a combination of power analysis based on pilot studies, limited by reagent availability, or expanded when additional animals were required to generate enough material (e.g. small lesions) for downstream analysis.
Data exclusions	Outliers were not excluded from the study. Animals were excluded from study if euthanized for non-study reasons in accordance with Animal Care and Use Guidelines.
Replication	Results were obtained by evaluating multiple animals per group (minimum 3). Results across replicates are reported in the Article, Supplementary Information, and Source Data file. Sequencing studies required animals to be pooled to obtain sufficient material for analysis and multiple pools of animals were evaluated, except for single cell sequencing studies where only a single pool was evaluated.
Randomization	Organisms were randomly allocated into experimental groups.
Blinding	Investigators were blinded during data collection and analysis during tumor palpation, tissue collection, and histological evaluation of (1) animals injected with indolent or aggressive tumor cells), and (2) animals treated with agent versus control. Investigators were also blinding during processing of post-digested samples isolated from indolent or aggressive lesions. Blinding was not possible in cases when group-specific features were evident during data collection or analysis of stain-positive cells in histologically-distinct lesions.

Reporting for specific materials, systems and methods

We require information from authors about some types of materials, experimental systems and methods used in many studies. Here, indicate whether each material, system or method listed is relevant to your study. If you are not sure if a list item applies to your research, read the appropriate section before selecting a response.

Materials & experimental systems

n/a	Involved in the study	
☐	☒	Antibodies
☒	☐	Eukaryotic cell lines
☒	☐	Palaeontology and archaeology
☐	☒	Animals and other organisms
☒	☐	Human research participants
☒	☐	Clinical data
☒	☐	Dual use research of concern

Methods

n/a	Involved in the study	
☒	☐	ChIP-seq
☐	☒	Flow cytometry
☒	☐	MRI-based neuroimaging

Antibodies

Antibodies used	CD19 (Thermo Fisher 14-0194-82); CD3 (Abcam ab5690); CD45 (e-Biosciences 14-0451-85); Col IV (Abcam ab6586); E-cadherin (Cell Signaling Technology 3195); ER-alpha (Millipore 06-935); F4/80 (Thermo MA1-91124); HA (BioLegend 901502); Ki67 (Abcam ab15580); MPO (Abcam ab208670); S100A8 (R&D Systems MAB3059). The following antibodies were used for imaging mass cytometry: CD163 (Abcam ab213612), conjugated to 168 Er; CK5 (Abcam ab214586), 145 Nd; CK8 (Abcam ab217173), 154 Sm; Col-IV (Abcam ab6586), 155 Gd; pS6 (CST 4858), 167 Er; S100A8 (R&D MAB3059), 115 In; Vimentin (Abcam 193555), 151 Eu, αSMA (CST D4K9N), 158 Gd; PE anti-mouse CD45 antibody, (BioLegend 103106).
Validation	CD19 (Thermo Fisher 14-0194-82), documented in https://www.citeab.com/antibodies/3517133-14-0194-cd19-monoclonal-antibody-6omp31-ebioscien?des=c5377abbc8b7d3ab, cited by 7<br>CD3 (Abcam ab5690), documented in https://www.abcam.com/cd3-antibody-ab5690, cited by 349<br>CD45 (e-Biosciences 14-0451-85), documented in https://www.thermofisher.com/antibody/product/CD45-Antibody-clone-30-F11-Monoclonal/14-0451-82, cited by 250<br>Col IV (Abcam ab6586), documented in https://www.abcam.com/collagen-iv-antibody-ab6586, cited by 568<br>E-cadherin (CST 3195), documented in https://www.cellsignal.com/products/primary-antibodies/e-cadherin-24e10-rabbit-mab/3195, cited by 1607<br>ER-alpha (Millipore 06-935), documented in https://www.citeab.com/antibodies/220600-06-935-anti-estrogen-receptor-alpha-antibody?des=0ae044017c3ca876, cited by 93

F4/80 (Thermo MA1-91124), documented in https://www.thermofisher.com/antibody/product/F4-80-Antibody-/MA1-91124, cited by 32
HA (BioLegend 901502), documented in https://www.biolegend.com/en-us/search-results/purified-anti-ha-11-epitope-tag-antibody-11374, cited by 371
Ki67 (Abcam ab15580), documented in https://www.abcam.com/ki67-antibody-ab15580, cited by 2282
MPO (Abcam ab208670), documented in https://www.abcam.com/myeloperoxidase-antibody-epr20257-ab208670, cited by 20
S100A8 (R&D Systems MAB3059), documented in https://www.citeab.com/antibodies/search?c=51&cl=1&q=s100a8&r=6809, cited by 8
CD163 (Abcam ab213612), 168 Er, documented in https://www.abcam.com/cd163-antibody-epr19518-bsa-and-azide-free-ab213612, cited by 2; https://www.abcam.com/cd163-antibody-epr19518-ab182422.html, cited by 108
CK5 (Abcam ab214586), 145 Nd, documented in https://www.abcam.com/cytokeratin-5-antibody-ep1601y-bsa-and-azide-free-ab214586.html, cited by 8; https://www.abcam.com/cytokeratin-5-antibody-ep1601y-cytoskeleton-marker-ab52635.html, cited by 81
CK8 (Abcam ab217173), 154 Sm, documented in https://www.abcam.com/cytokeratin-8-antibody-ep1628y-bsa-and-azide-free-ab217173.html, cited by 14; https://www.abcam.com/cytokeratin-8-antibody-ep1628y-cytoskeleton-marker-ab53280.html, cited by 66
Col-IV (Abcam ab6586), 155 Gd, documented in https://www.abcam.com/collagen-iv-antibody-ab6586.html, cited by 568
pS6 (CST 4858), 167 Er, documented in https://www.cellsignal.com/products/primary-antibodies/phospho-s6-ribosomal-protein-ser235-236-d57-2-2e-xp-rabbit-mab/4858, cited by 798
S100A8 (R&D MAB3059), 115 In, documented in https://www.citeab.com/antibodies/search?c=51&cl=1&q=s100a8&r=6809, cited by 8
Vimentin (Abcam 193555), 151 Eu, documented in https://www.abcam.com/vimentin-antibody-epr3776-bsa-and-azide-free-ab193555.html, cited by 34; https://www.abcam.com/vimentin-antibody-epr3776-cytoskeleton-marker-ab92547.html, cited by 872
αSMA (CST 19245), 158 Gd, documented in https://www.cellsignal.com/products/primary-antibodies/a-smooth-muscle-actin-d4k9n-xp-rabbit-mab/19245, cited by 116
PE-CD45 (BioLegend 103106), documented in https://www.biolegend.com/en-us/products-2/pe-anti-mouse-cd45-antibody-100, cited by 73

Animals and other organisms

Policy information about studies involving animals; ARRIVE guidelines recommended for reporting animal research

Laboratory animals	Mus musculus, FVB/NJ Stain 001800, females, adult (aged 12-15 weeks)
Wild animals	This study did not involve wild animals.
Field-collected samples	This study did not involves samples collected from the field.
Ethics oversight	Animal work described was performed in accordance with recommendations in the Guide for Care and Use of Laboratory Animals from the National Institutes of Health (NIH) Institutional Animal Care and Use Committee (IACUC) and the American Association for Accreditation of Laboratory Animal Care (AAALAC). Studies were approved by the University of Texas MD Anderson Cancer Center IACUC.

Note that full information on the approval of the study protocol must also be provided in the manuscript.

Flow Cytometry

Plots

Confirm that:

- [x] The axis labels state the marker and fluorochrome used (e.g. CD4-FITC).
- [x] The axis scales are clearly visible. Include numbers along axes only for bottom left plot of group (a 'group' is an analysis of identical markers).
- [x] All plots are contour plots with outliers or pseudocolor plots.
- [x] A numerical value for number of cells or percentage (with statistics) is provided.

Methodology

Sample preparation

Resected mammary glands bearing caErbB2-GFP lesions were visualized under a fluorescence stereoscope, and regions harboring aggressive (>2mm, comprised of invasive lesions) and indolent (<2mm, enriched for in situ lesions) lesions were dissected away from each other. Tissues were mechanically minced, and then digested to organoids in epithelial cell media [advanced DMEM/F12 (Gibco) with 5% fetal bovine serum (Hyclone), and 1% antibiotic-antimycotic (Corning)], supplemented with 3 mg/ml collagenase (Roche), 0.6 mg/ml hyaluronidase (Sigma), 1.3% bovine serum albumin (BSA) (Sigma). Tissue digests were agitated by angled rotation for 2-5 hours at 37°C, pelleted by centrifugation at 450g, and then resuspended in red cell lysis buffer (Sigma). Following wash with epithelial cell media and PBS, pelleted organoids were incubated in 0.25% Trypsin-EDTA (Corning) at room temperature for 5 minutes. Cells were again washed, pelleted, and resuspended in epithelial cell media supplemented with up to 10U dispase (Stem Cell) and 5ug DNAase I (Stem Cell), filtered serially through 100 μm and 70 μm cell strainers (Falcon), and enumerated by trypan blue staining. Following digestion of tissues down to single cells, samples were stored at 4°C overnight in Hypothermosol (BioLife Solutions). The following day, tumor cells were washed and

resuspended in [PBS with 0.5% EDTA] for sorting.

Instrument	BD FACSAria
Software	BD FACSDiva
Cell population abundance	Samples were visualized immediately post-sort to confirm that sorted population expressed GFP. Samples sorted for CD45+ immune cells contained the intended population, while samples sorted for GFP+ tumor cells included some stromal cell contaminants, as determined by single cell analysis.
Gating strategy	Cells were gated on forward- versus side-scatter areas for cell size, then forward-scatter area versus height and side-scatter area versus height for single cells, and then by fluorophore for gene expression.

☒ Tick this box to confirm that a figure exemplifying the gating strategy is provided in the Supplementary Information.
